# Spectroscopic/Bond Property Relationship in Group 11 Dihydrides via Relativistic Four-Component Methods

**DOI:** 10.1021/acs.jpca.0c09043

**Published:** 2020-12-02

**Authors:** Diego Sorbelli, Matteo De Santis, Paola Belanzoni, Leonardo Belpassi

**Affiliations:** † Department of Chemistry, Biology and Biotechnology, University of Perugia, via Elce di Sotto 8, 06123 Perugia, Italy; ‡ CNR Institute of Chemical Science and Technologies “Giulio Natta” (CNR-SCITEC), c/o Department of Chemistry, Biology and Biotechnology, University of Perugia, 06123 Perugia, Italy; § Consortium for Computational Molecular and Materials Sciences (CMS)2, via Elce di Sotto 8, 06123 Perugia, Italy

## Abstract

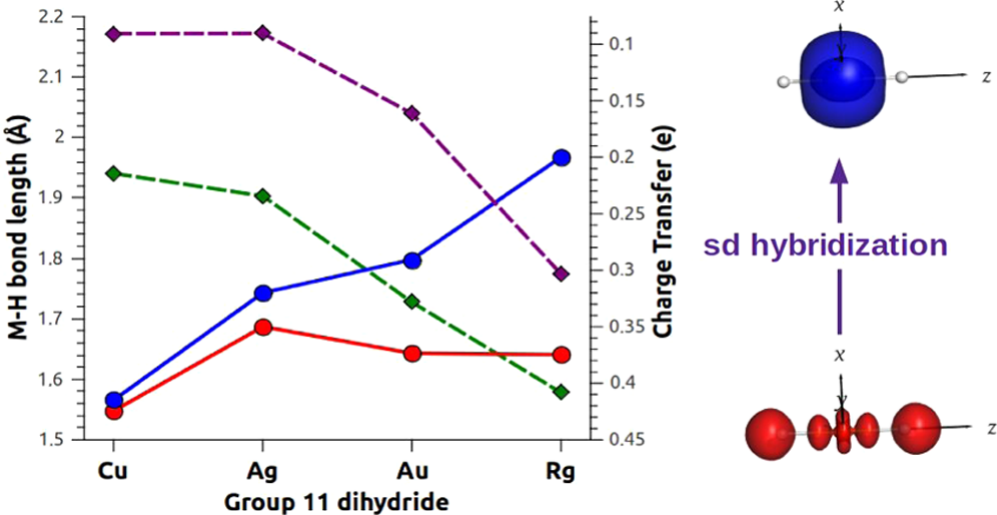

Group 11 dihydrides MH_2_^–^ (M = Cu, Ag, Au, Rg) have been much less studied than the corresponding MH compounds, despite having potentially several interesting applications in chemical research. In this work, their main spectroscopic constants (bond lengths, dissociation energies, and force constants) have been evaluated by means of highly accurate relativistic four-component coupled cluster (4c-CCSD(T)) calculations in combination with large basis sets. Periodic trends have been quantitatively explained by the charge-displacement/natural orbitals for chemical valence (CD-NOCV) analysis based on the four-component relativistic Dirac–Kohn–Sham method, which allows a consistent picture of the nature of the M–H bond to be obtained on going down the periodic table in terms of Dewar–Chatt–Duncanson bonding components. A strong ligand-to-metal donation drives the M–H bond and it is responsible for the heterolytic (HM···H^–^) dissociation energies to increase monotonically from Cu to Rg, with RgH_2_^–^ showing the strongest and most covalent M–H bond. The “V”-shaped trend observed for the bond lengths, dissociation energies, and stretching frequencies can be explained in terms of relativistic effects and, in particular, of the relativistically enhanced sd hybridization occurring at the metal, which affects the metal–ligand distances in heavy transition-metal complexes. The *sd* hybridization is very small for Cu and Ag, whereas it becomes increasingly important for Au and Rg, being responsible for the increasing covalent character of the bond, the sizable contraction of the Au–H and Rg–H bonds, and the observed trend. This work rationalizes the spectroscopic/bond property relationship in group 11 dihydrides within highly accurate relativistic quantum chemistry methods, paving the way for their applications in chemical bond investigations involving heavy and superheavy elements.

## Introduction

Copper, silver, and gold hydrides (MH compounds, otherwise referred to as “coinage metal’ hydrides” and hereafter also reported as “monohydrides”) have been known for more than a century (the first example of polymeric Cu(I) hydride is from the 1840s),^1^ and nowadays, there is plenty of literature concerning their characterization.^2−9^ The great interest for these compounds is motivated by the range of applications they have in chemical research, such as in homogeneous catalysis^10^ or in the field of renewable energies, where they could be used for hydrogen storage.^11−14^ In addition, the study of the M–H bond in these species is highly appealing for the exploration of relativistic effects in chemistry. Indeed, gold has been identified as the element where a local maximum of relativistic effects is observed^15^ and therefore coinage metal hydrides have often been used for analyzing in detail the role of relativistic effects along a group of the periodic table.^15−20^

The relevance of using these species as probes for relativistic effects grew even more after 1994, when the heavier homologue of gold, roentgenium (Rg), was artificially synthesized.^21^ As roentgenium is a superheavy element, relativistic effects (scalar and spin–orbit coupling) are expected to have a huge impact on its chemical behavior, even to a greater extent with respect to gold. However, Rg is not stable enough to let its compounds being experimentally investigated (the half-lives of its isotopes range from milliseconds to a maximum of a few minutes). Therefore, the only viable way to explore the chemical behavior of this superheavy element is through theoretical calculations, which must include the relativistic effects (and electron correlation) at the highest accuracy. In particular, Rg hydrides are the smallest molecular compounds containing the Rg atom and constitute an appropriate playground for exploring relativistic effects in a molecular framework, where the most accurate relativistic quantum chemistry methods can effectively be applied. Suitable quantities for the analysis of the chemical bond and relativistic effects on coinage metals’ monohydrides can be spectroscopic constants, such as equilibrium bond lengths, dissociation energies, and force constants (or vibrational frequencies, which can be directly measured in the vibrational spectroscopy). They allow to quantitatively get insights into the strength and nature of the M–H chemical bond. Currently, several theoretical studies about the RgH molecule can be found in the literature, where a large number of them focus on the computation of the spectroscopic constants of RgH in comparison with those of its lighter homologues.^22−27^

Although the chemical properties of group 11 monohydrides have been intensively explored throughout the years, the same is not true for group 11 dihydrides (MH_2_^–^, M = Cu, Ag, Au, Rg).

In principle, these compounds have the same potential with respect to the monohydrides, since they can be used for the basic understanding of the metal–hydrogen bond formation which is important for catalytic and energy-related purposes, as discussed to some extent by Andrews et al.^28^ Similarly to monohydrides, these molecules are simple enough to be investigated via four-component relativistic approaches. Nevertheless, the available information about this class of compounds is much more scarcer than that for the corresponding monohydrides. Experimental characterization lacks mainly due to the difficulties in generating these species, and the chemistry of these complexes has not been largely discussed from a theoretical perspective either. One of the few examples in which CuH_2_^–^, AgH_2_^–^, and AuH_2_^–^ have been experimentally characterized consists of an infrared spectroscopy study in which the systems are trapped in a solid matrix. In the same study, a theoretical analysis of their equilibrium geometries and the mechanism of formation of these complexes has also been given.^28^ Furthermore, the gas-phase reactivity of copper dihydride was discussed to some extent^29^ and gold dihydride has been investigated by the means of photoelectron spectroscopy.^30^ Bonding and relativistic effects in AuH_2_^–^ have been discussed from a theoretical perspective in few studies.^30−32^ Particularly noticeable is the work by Xu et al., where the periodicity and covalency of the M–H bond in MH_2_^–^ complexes have been discussed based on density functional theory (DFT) calculations, including for the first time, to the best of our knowledge, RgH_2_^–^ in the dissertation.^33^

All of these available studies point out that the M–H bond in these complexes is mainly driven by ligand-to-metal σ electron charge donation. Intriguing features also emerge from the analysis of the M–H bonding, such as the population of the hydrogen 2p atomic orbitals of H via π back-donation from the metal. Moreover, the sd_*z*^2^_ hybridization that occurs at the metal is also expected to play a role, especially for Au and Rg. All of these features are certainly highly dependent on relativistic effects and a periodicity can be found, with RgH_2_^–^ having the strongest and most covalent bond in the group 11 series. In addition, the features of the Rg–H bond have been shown to be significantly influenced, as it could be expected, by spin–orbit coupling.

This framework provides a particularly interesting and qualitatively defined picture of bonding in these complexes. The focus of this work is, however, to analyze bonding from a different and more quantitative perspective. It was mentioned earlier that, upon the discovery of Rg, several studies dealing with the theoretical estimation of the spectroscopic constants in group 11 monohydrides, their quantitative evaluation, and the impact of relativistic effects on them have appeared in the literature. Unfortunately, similar systematic and theoretically highly rigorous investigations have not been carried out for MH_2_^–^ compounds yet. The geometries of MH_2_^–^ molecules and the force constants of the M–H bond were previously calculated at the DFT level and with approximate relativistic approaches.^28,33^ However, as it has been previously highlighted, an accurate inclusion of both relativistic effects and electron correlation can enormously impact the results for monohydrides^25^ and it is reasonable to expect a similar effect on dihydrides.

For this reason, equilibrium M–H bond lengths and dissociation energies have been calculated for MH_2_^–^ compounds using the highly accurate relativistic 4c-CCSD(T) in conjunction with extended basis sets. The same method has been used for estimating the force constants and the stretching frequencies of this series of dihydrides, in tight comparison with the experimental results for coinage metal dihydrides previously reported.^28^ These spectroscopic constants are determined in this work with high accuracy, and it is shown that well-defined trends are present.

A detailed analysis of the chemical bond has been carried out by the means of the so-called charge displacement (CD) via natural orbitals for chemical valence (CD-NOCV) method. In particular, we apply a recent implementation that extends the applicability of this approach to the relativistic four-component framework. With this methodology, we are able to give a reliable quantitative measure of the donation and back-donation components of the bond and thus to assess the nature of the bond character with the most accurate inclusion of relativistic effects and spin–orbit coupling. Furthermore, a detailed analysis of the spatial extent of NOCV spinors allows a quantitative picture of the charge rearrangement due to sd hybridization to be given. We thus quantify both the degree of covalency and the sd hybridization in the group 11 dihydrides in tight connection with the calculated spectroscopic constants.

## Methods and Computational Details

Geometry optimizations, heterolytic dissociation energy (HM–H^–^) and harmonic frequency calculations have been carried out at the CCSD(T) level using the four-component Dirac–Coulomb Hamiltonian (4c-CCSD(T)) as implemented in the DIRAC code (in its 2018 release).^34,35^ In all calculations, Dyall’s valence triple-ζ quality basis set was used.^36−39^ In all cases, *n*s, *n*p, *n*d, and (*n* + 1)s electrons have been correlated (with *n* = 3 for Cu, *n* = 4 for Ag, *n* = 5 for Au, and *n* = 6 for Rg). The heterolytic dissociation energies have been calculated by considering the following dissociative mechanism
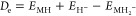
1where *E*_MH_2_^–^_ refers to the energy of the relaxed anionic dihydride, *E*_MH_ to the energy of the relaxed monohydride fragment, and *E*_H^–^_ to the energy of the isolated hydride.

Selected cuts of the potential energy surfaces (PESs) of the four dihydrides have also been carried out at the same level of theory by keeping one M–H bond length fixed and varying the other.

The approach we selected for the optimizations is described in the Supporting Information (SI). Geometry optimizations have also been carried out using a nonrelativistic Hamiltonian. The force constants necessary for the calculation of the numerical asymmetric and symmetric H–M–H stretching frequencies have been obtained by single-point calculations where one M–H bond length is kept fixed and the other varied. The force constants (i.e., the elements of the Hessian matrix) have then been calculated with the numerical procedure detailed in SI with the same four-component relativistic protocol. The heterolytic dissociation energies have also been calculated with Dyall’s double- and quadruple-ζ valence basis sets, and we used the following two-point extrapolation scheme reported by Helgaker et al.^40,41^ for estimating the energies at the basis set limit
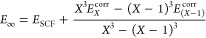
2with *X* being the cardinal number of the correlation consistent basis set.

Geometry optimizations have also been carried out for comparison at the DFT level with several GGA (PBE,^42^ BLYP,^43,44^ BP86^43,45^), hybrid (PBE0,^46,47^ B3LYP,^48^ S12H^49^), meta-GGA (M06-L,^50^ TPSS^51^), and double hybrid (M06,^52^ TPSSh^53^) exchange–correlation functionals. The ADF program code was used (2014 version).^54^ In all cases, calculations were performed with no frozen core approximation (all electron) and a Slater-type basis set of quadruple-ζ quality with polarization functions (QZ4P). Relativistic effects were introduced through the approximate two-component scalar (SR) zero-order relativistic approximation (ZORA) Hamiltonian^55−57^ and spin–orbit (SOC) ZORA Hamiltonian,^58,59^ for including scalar relativistic effects and spin–orbit coupling, respectively. The same computational setup was used for the calculations of interaction energies.

For the study of the M–H bond, we applied the charge-displacement (CD) analysis via natural orbital for chemical valence (NOCV) recently developed in the four-component relativistic framework. The CD approach is a powerful tool for the analysis of bonding that allows us to measure the exact amount of electron density that, upon the formation of a bond between two fragments, is transferred from a fragment to another. In general, CD analysis has been previously used to characterize interactions between noble gases and Au,^60^ adducts with hydrogen or halogen bonds,^61−63^ organometallic complexes,^64−67^ and electronic excited states.^68^ The CD function (Δ*q*) is defined as the partial progressive integration on a suitable *z*-axis of the electron density difference (Δρ) between the density of the adduct and the sum of the densities of the noninteracting fragments at the positions they have in the adduct geometry^60^
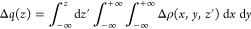
3In eq 3, the integration axis is conveniently chosen as the bond axis between two fragments constituting the adduct. In this work, if not explicitly stated differently, the fragments are the HM molecule (with M = Cu, Ag, Au, Rg) and the H^–^ anion.

The CD function, Δ*q*(*z*), quantifies at each point of the bond axis the exact amount of electron charge that, upon the formation of the bond, is transferred from the right to the left across a plane perpendicular to the bond axis through *z*. To be able to measure charge transfer (CT) in this context, we need to take the CD value at some specific point between the fragments (i.e., at an arbitrarily defined plane separating them). The usual choice, which is at the so-called “isodensity boundary”, is the *z*-point where equally valued isodensity surfaces of the isolated fragments become tangent.^69^

When describing the coordination bond in transition-metal complexes, it can be useful to refer to the Dewar–Chatt–Duncanson (DCD) components for its description (i.e., σ donation and π back-donation). The CD scheme reported in eq 3 does not provide a partition of the density rearrangements into these chemically relevant components. This can be achieved by relying on the theory of NOCV, introduced by Mitoraj and Michalak^70,71^ as descriptors of a chemical bond. This approach is suitable for the description of chemical bonding since the electron density difference can be brought into diagonal contributions in terms of NOCVs.

In the NOCV scheme, the charge rearrangement taking place upon bond formation is obtained from the occupied orbitals of the two fragments suitably orthogonalized to each other and renormalized (“promolecule”). The resulting electron density rearrangement can be expressed in terms of NOCV pairs, which are defined as the eigenfunctions of the so-called “valence operator”^72−74^ as follows
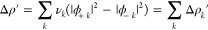
4where ϕ_+*k*_ and ϕ_–*k*_ are the NOCV pair orbitals and *v*_±*k*_ are the eigenvalues. When the adduct is formed from the promolecule, a fraction ν*_k_* of electrons is transferred from the ϕ_–*k*_ to the ϕ_+*k*_ orbital. The CD and NOCV schemes can be effectively coupled, resulting in the so-called CD-NOCV scheme.^75^ Since only few of the NOCV pairs contribute to the actual chemical bond, when the CD-NOCV analysis is performed, usually the first Δρ_*k*_′ components are investigated to understand which significant chemical contribution to the bond they represent.

Since the CD-NOCV theory is based on a very general formulation, one can perform this analysis in a relativistic four-component framework. In this case, the relativistic NOCVs are obviously four-component vectors, i.e., spinors (they can be referred to as “natural spinors for chemical valence”). In this work, we use the four-component CD-NOCV scheme^76^ as implemented in the Dirac–Kohn–Sham module of the BERTHA code.^77−82^ This approach represents the state-of-the-art technique to include the spin–orbit coupling effect in the bonding analysis,^76^ since it allows the CD analysis at a four-component Dirac–Kohn–Sham level to be performed. This is of particular interest for compounds containing heavy and superheavy elements, where relativistic effects and spin–orbit coupling may play a crucial role in the description of bonding. Computational details for this approach have been reported in the Supporting Information.

We also calculated the populations of the metals’ atomic orbitals by relying on the projection analysis (PA).^83−85^ This analysis is based on the linear combination of atomic orbitals (LCAO) approximation, according to which a molecular orbital (|ψ_*i*_^MO^⟩) can be decomposed into contributions from orbitals (*p*) of the atom (or the fragment) *A* as follows
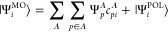
5where *c_pi_^A^* represents the expansion coefficients and the last term is denoted the polarization contribution. Typically, the occupied orbitals of the constituent atoms are selected. However, they do not necessarily fully span the molecular orbitals and for this reason the expansion in eq 5 includes their orthogonal complement, i.e., |ψ_*i*_^POL^⟩. In our case, to gain the highest possible accuracy, we selected all occupied and virtual orbitals of the constituent atoms (thus, the polarization contribution is zero). By projection with |ψ_*q*_^*B*^⟩ (where *B* represents the atom/fragment index and *q* the corresponding orbitals’ index), the expansion coefficients *c*_*pi*_^A^ are found by solving the equation
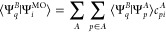
6The analysis is practically basis set independent,^84^ and it can be used in a four-component framework. Moreover, localization procedures for the molecular orbitals can be applied, making PA a strong and reliable tool for the analysis of chemical bonding in a four-component framework. In this case, the analysis has been carried out with the DIRAC code both in the nonrelativistic and in the four-component Dirac–Kohn–Sham frameworks, using Dyall’s triple-ζ basis set and the PBE functional.

For testing purposes, we have also employed the CD-NOCV scheme using scalar relativistic (SR) Hamiltonian within the ZORA as implemented in the ADF code.^54^ The SR-ZORA Hamiltonian was used with no frozen core approximation (all electron) and a Slater-type basis set of quadruple-ζ quality.

## Results and Discussion

### Spectroscopic and Structural Properties

The first part of this work is devoted to the evaluation of the spectroscopic constants of the MH_2_^–^ (M = Cu, Ag, Au, Rg) complexes via highly accurate four-component relativistic approaches.

As mentioned, some information is already known about the equilibrium M–H bond lengths for these complexes from the literature. These systems are known to be linear with a centrosymmetric structure. A very accurate reference is available for the Au–H bond length in AuH_2_^–^,^30^ while DFT calculations can be found for the other MH_2_^–^ complexes.^31,33^ We already pointed out that an accurate inclusion of both relativistic effects (including spin–orbit coupling) and electron correlation affects bond lengths in MH compounds,^25^ and it is reasonable to expect the same impact for MH_2_^–^ complexes. First, we carried out several DFT geometry optimizations for AuH_2_^–^ by varying both the functional and the relativistic Hamiltonian (i.e., scalar and spinorbit ZORA Hamiltonians) (Tables S1 and S2 in the SI), showing that the results only slightly depend on both the functional and the relativistic Hamiltonian used for the calculations.

However, to avoid any dependency on the specific functional employed and/or on the approximated Hamiltonian and with the aim of achieving a very high degree of accuracy, 4c-CCSD(T) calculations have been carried out. This method has been at first validated by calculating the M–H bond lengths for monohydrides. The M–H distances have been experimentally measured for coinage metal monohydrides^86^ and these values have been considered as a reference for performing a first evaluation of the level of accuracy we can achieve with our approach. The results (reported in Table S3 in the SI) show that the method is extremely accurate for coinage metal monohydrides and yields results that are consistent with the ones previously calculated for RgH.^23−25,27,87^ On this basis, equilibrium M–H bond lengths in MH_2_^–^ complexes have been optimized with a 4c-CCSD(T) approach. Nonrelativistic calculations have also been carried out to get a quantitative and accurate evaluation of the extent of the contraction of the M–H bond length due to relativistic effects (relativistic bond contraction, RBC). This quantity has been helpful in the past for evaluating the effect of relativity in contracting the bond length in heavy metals’ diatomic compounds and, in particular, in group 11 hydrides, showing that for AuH and RgH, relativistic effects heavily contract the bond lengths, with the contraction being roughly proportional to the square of the atomic number of the metal (*Z*^2^).^15,22^

**Figure 1:**
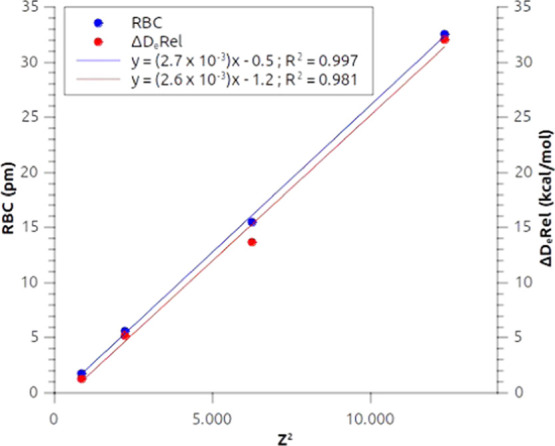
Correlation between the square of the atomic number (*Z*^2^) of the group 11 metal (M) and the relativistic bond contraction (RBC, blue) and the relativistic increase of the dissociation energy (ΔD_e_Rel, red) of the M–H bond in MH_2_^–^ complexes.

In Table 1, the 4c-CCSD(T) calculated bond length values (*R*_M–H_^4c^) for MH_2_^–^ complexes are reported, together with the corresponding RBC. As expected, a contraction due to relativity can be observed, which is practically negligible for Cu and becomes increasingly important on descending along the group (for RgH_2_^–^ the bond contraction is almost 20%). This trend is already well-known for group 11 monohydrides (as reported in ref (25) and in Figure S1 in the SI). Our calculations unravel a very similar behavior for MH_2_^–^ complexes: the bond contraction induced by relativistic effects is roughly proportional to *Z*^2^, as shown in Figure 1.

**Table 1:** 4c-CCSD(T)-Calculated Equilibrium M–H Distances (*R*_M–H_) and M–H Heterolytic Dissociation Energies (*D*_e_) for the MH_2_^–^ Complexes (M = Cu, Ag, Au, Rg)^a^

spectroscopic and structural properties MH_2_^–^	*R*_M–H_ (Å)	RBC (pm)	*D*_e_ (kcal/mol)	ΔD_e_Rel (kcal/mol)
Cu	1.548 (1.566)	1.8	79.76 (78.13)	1.32
Ag	1.687 (1.743)	5.6	78.44 (73.19)	5.25
Au	1.643 (1.798)	15.5	90.86 (77.19)	13.67
Rg	1.641 (1.967)	32.6	99.55 (67.49)	32.06

a The corresponding nonrelativistic values are also reported in parenthesis. The relativistic bond contraction (RBC) and the relativistic increase of the dissociation energy (ΔD_e_Rel) are shown for each complex. The dissociation energies have been calculated as described in eq 1.

The bond length variation along the group exhibits the following trend for group 11 dihydrides: Cu–H < Rg–H < Au–H < Ag–H. Noticeably, SR-DFT calculations reported in ref (33) predicted a different trend (Cu–H < Au–H < Ag–H ≈ Rg–H). We already briefly discussed, however, about DFT results variability depending on the level at which relativistic effects are introduced and on the functional used. Moreover, the trend found here for dihydrides is very similar to that observed both previously^25^ and in the current study for monohydrides (Table S3 in the SI) and also resembles the trend observed for atomic radii^88,89^ of group 11 metals, which suggests the strong relativistic stabilization of 6s and 7s orbitals to be responsible for the very short Au–H and Rg–H distances, respectively. The same trend has also been observed in a recent work for the M–M distances in group 11 dimers M_2_ (M = Cu, Ag, Au, Rg) (Cu < Rg < Au < Ag), where correlated approaches have been used to investigate their spectroscopic constants.^90^ The differences between DFT and 4c-CCSD(T) results point out that a very accurate treatment of electron correlation and relativistic effects is a particularly important ingredient when dealing with these properties.^25^

In addition, the above results suggest that the Rg–H bond in RgH_2_^–^ may be the strongest bond in the series, having a very short M–H bond length. A more quantitative perspective on the strength of the M–H bond can be obtained by computing the dissociation energies of MH_2_^–^ complexes. Based on the literature, these anionic compounds are formed via the following associative mechanism^28^
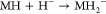
7For this reason, we calculate these energies by considering the heterolytic dissociation of the dihydrides into the two MH and H^–^ fragments. DFT predicts interaction energies that depend on the specific approximation of the exchange–correlation functional and, in particular, on the relativistic Hamiltonian used (as reported in Table S4 in the SI). The heterolytic dissociation energies for group 11 dihydrides computed at the reference 4c-CCSD(T) level are reported in Table 1. We observe a very clear trend that agrees with the one found for bond lengths: dissociation energies show a minimum for AgH_2_^–^ and then increase up to RgH_2_^–^, which displays the highest dissociation energy (about 100 kcal/mol). Moreover, *D*_e_ values show a large variability, spanning a range of about 30 kcal/mol. We also performed a basis set extrapolation to the infinite basis set limit with the scheme reported in the Methods and Computational Details section, where the very accurate values we obtain confirm these trends (see Table 2).

**Table 2:** 4c-CCSD(T)-Calculated Heterolytic Dissociation Energies (*D*_e_) for MH_2_^–^ (M = Cu, Ag, Au, Rg) Complexes with Different Dyall’s Valence Basis Sets^a^

	*D*_e_ (kcal/mol)			
MH_2_^–^	dyall.vdz	dyall.vtz	dyall.vqz	dyall.v∞z
Cu	83.37	79.76	78.82	78.97
Ag	83.56	78.44	74.54	72.61
Au	93.50	90.86	89.37	89.06
Rg	99.26	99.55	98.74	98.94

a The dissociation energies have been calculated as described in eq 1. Dyall.v∞z refers to the basis set extrapolation using the expression reported in eq 2 with *X* = 4.

Several features of these trends deserve a discussion. First of all, by computing dissociation energies at the nonrelativistic CCSD(T) level, we find that a relativistic increase (ΔD_e_Rel) affects them and, analogously to bond lengths, this quantity is roughly proportional to *Z*^2^ (Figure 1). By comparing our results with those we find in the literature, we see that this dissociation energy trend is well-known and somehow expected for these complexes. A similar trend is also displayed for group 11 dimers^90^ and an analogous trend with the Ag–H showing the lowest value of *D*_e_ is also reported for group 11 monohydrides.^25^

A different perspective for estimating the strength of the M–H bond is to compute the M–H force constants and the stretching frequencies, which, in the case of coinage metal dihydrides, can be directly compared to the experimental values.^28^ 4c-CCSD(T) calculated force constants and numerical stretching frequencies are reported in Table 3.

**Table 3:** 4c-CCSD(T)-Calculated Numerical Symmetric (υ̃^symm^) and Antisymmetric (υ̃^antisymm^) H–M–H Stretching Frequencies for MH_2_^–^ (M = Cu, Ag, Au, Rg) Complexes^a^

MH_2_^–^	υ̃^symm^ (cm^–1^)	υ̃^antisymm^ (cm^–1^)	υ̃_Ne_^exp^ (cm^–1^)	υ̃_Ar_^exp^ (cm^–1^)	υ̃_H2_^exp^ (cm^–1^)	*k*_M–H_ (N/m)
Cu	1730.8	1575.1	1529.5	1497.2	1517.8	143.6
Ag	1663.5	1512.4	1460.0	1427.5	1442.4	130.9
Au	2018.9	1699.2	1638.6		1636.0	170.7
Rg	2285.9	1911.8				214.6

a The experimental antisymmetric stretching frequencies (υ̃^exp^), obtained in excess of Ar, Ne, and H_2_ from ref (28), are reported for comparison. The calculated adiabatic force constants of the M–H bond (*k*_M–H_) are also shown.

First, we see that the antisymmetric stretching frequencies we calculate here for coinage metal dihydrides are close to the experimental ones, despite systematically overestimating them. The systematic shift may be due to the fact that harmonic frequencies are computed, and anharmonicity may play an important role. Moreover, it should be noted that the reference experimental measurements are performed in different environments and the matrix effects are estimated to be responsible for a red shift of 5–20 cm^–1^ of the actual frequencies.^30^ In Table 3, we report for comparison the measurements in excess of H_2_, Ar, and Ne. As it can be seen, changing the environment can modify the frequencies up to 32 cm^–1^. Based on these considerations, we believe that the combination of these two effects may have an impact on the results, with the actual frequencies being slightly blue-shifted. However, in general, the approach we use is very accurate (see also a discussion in the SI) and useful for analyzing the stretching modes of RgH_2_^–^, for which, being a superheavy element, a rigorous four-component approach is clearly mandatory for an accurate prediction of these quantities.

The trend displayed for both stretching frequencies and M–H force constants appears, with no surprises, to be the same trend as that observed for dissociation energies, with the Ag–H bond showing the lowest force constant (and stretching frequencies) and Rg–H the highest. The same trend has been observed in the case of the group 11 dimers,^90^ where the sharp increase of both dissociation energies and force constants in Au_2_ and Rg_2_ has been ascribed to the relativistic increase of the extent of sd_*z*^2^_ hybridization, which should cause the bond to be shortened and strengthened. We will return to this point later.

**Figure 2:**
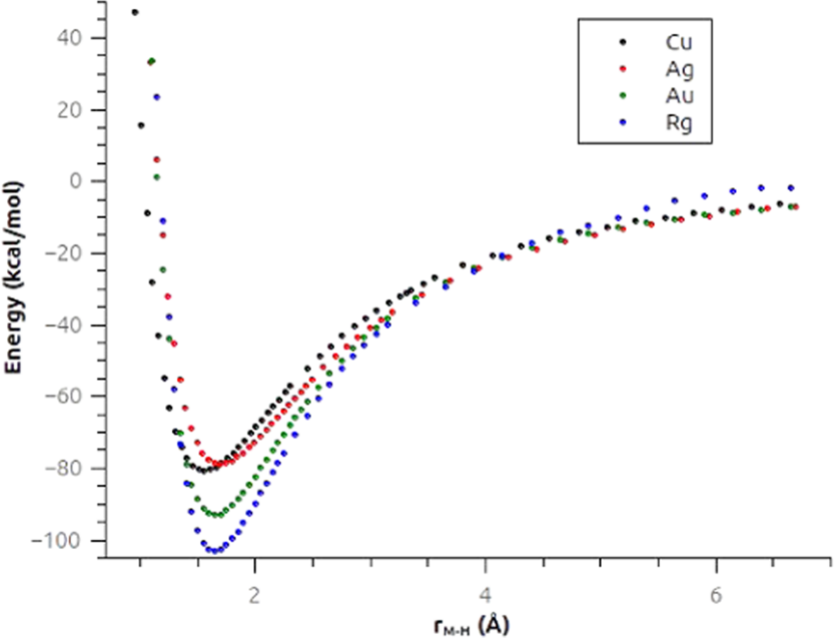
Selected cuts of the H–M···H^–^ potential energy surfaces (PESs) for the group 11 dihydrides calculated at the 4c-CCSD(T) level. The PESs have been calculated by keeping one M–H bond length fixed and varying the other. The energy has been shifted in each case according to the infinite M–H distance.

To summarize, we characterized with high accuracy the main structural and spectroscopic features of the M–H bond in these complexes, showing that for both Au and Rg relativistic effects deeply influence the nature of the M–H bond. Moreover, the Rg–H bond appears to be the strongest along the series. The PESs reported in Figure 2 show an additional peculiarity of the Rg containing system. Indeed, the 4c-CCSD(T)-calculated curves show features we already noticed, such as the equilibrium bond length and dissociation energy trends (that can be estimated by considering the positions of the minima of the curves). In the case of RgH_2_^–^, however, we also see that the Rg–H short-range interaction differs consistently from the long-range one. From the region of the curve in the proximity of the potential well, as expected from the results we discussed earlier, we see that the HRg–H^–^ short-range interaction is strong, leading us to expect the Rg–H bond to be characterized by a high degree of CT (covalency), consistently with literature results.^33^ However, in the long-range region of the PES (say above 4 Å), we see that the weakest long-range interaction among MH_2_^–^ compounds can be predicted for RgH_2_^–^. This is perfectly consistent with the inverted dipole reported for the RgH fragment, where a partial negative charge is expected to be located on Rg.^22,23^ Therefore, this long-range weak interaction (which differs qualitatively from the other group 11 metals) is due to the long-range electrostatic repulsion. This is consistent with what happens with the PESs evaluated using the nonrelativistic Hamiltonian (Figures S2–S5 in the SI), where no inversion of the dipole^22^ for the RgH molecule occurs and therefore the long-range interaction is very similar for all systems.

In the next section, we will try to rationalize and interpret the trends observed above for the spectroscopic constants by giving a quantitative measure of the electronic density rearrangement that is involved in the M–H bond formation (covalency) and that occurs at the metal site (sd hybridization mechanism). Both features are expected to increase due to the relativistic effects on going down the coinage metal series.

### Bonding in MH_2_^–^ Complexes: CD-NOCV Analysis

In this section, we aim at characterizing the M–H bond in the series of the MH_2_^–^ complexes with M = Cu, Ag, Au, and Rg. In particular, we provide a quantitative assessment of the Dewar–Chatt–Duncanson (DCD) bonding components (ligand-to-metal σ donation and metal-to-ligand π back-donation) and how these are tuned by the relativistic effects, which become increasingly important on going down the periodic table. We base our analysis on the CD-NOCV methodology, recently extended to the relativistic four-component framework (see the “Methods and Computational Details” section), implemented in the code BERTHA, which has been recently used for successfully characterizing the chemical bond involving heavy metals, such as in MCN complexes (M = coinage metals),^91^ and the impact of the spin–orbit coupling on the halogen bond involving astatine.^92^

**Figure 3:**
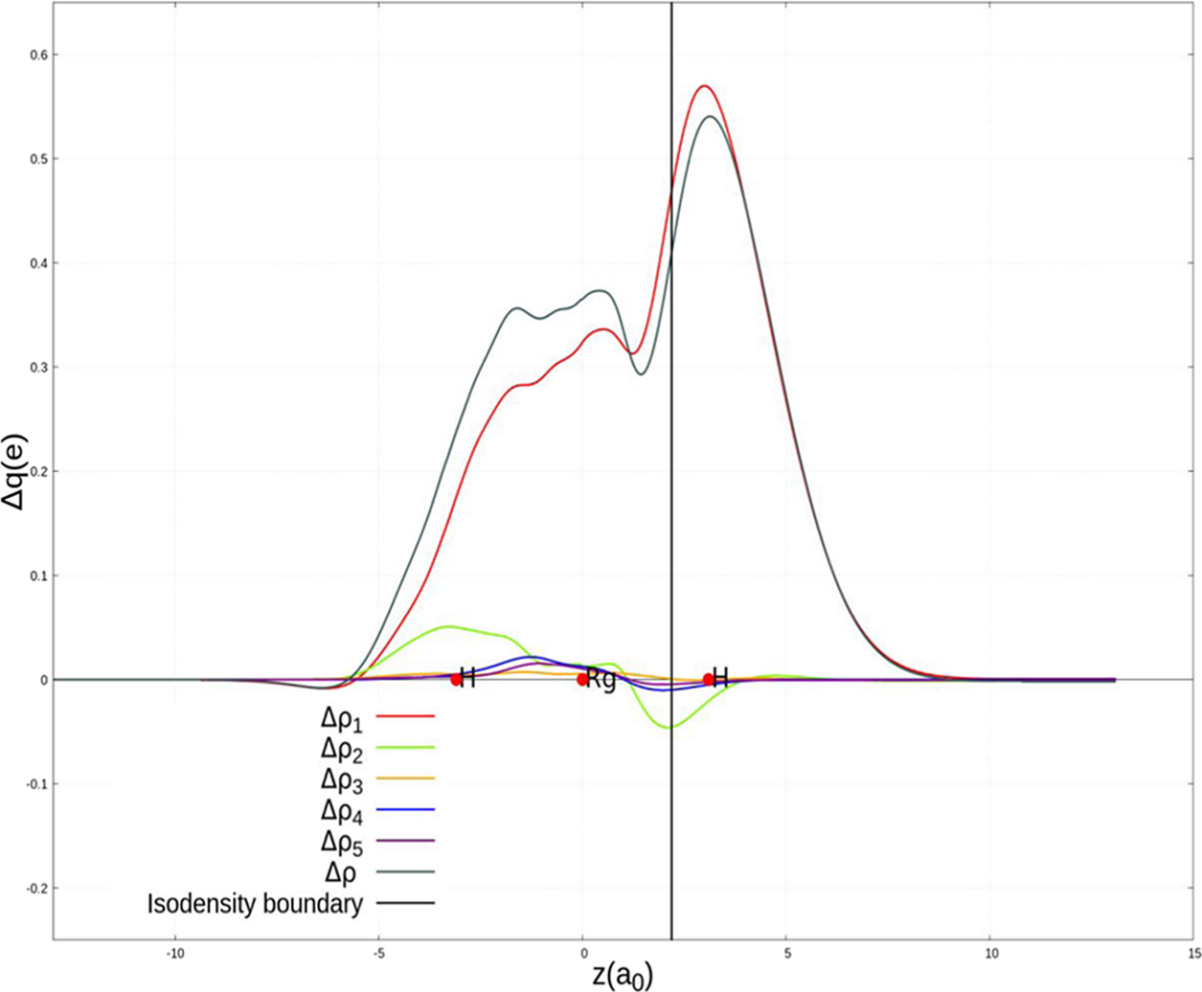
Charge-displacement (CD) curves for the RgH_2_^–^ complex. Red dots indicate the position of the nuclei along the *z*-axis. The vertical line marks the isodensity boundary between the HRg and H^–^ fragments (see the text for details). Distances are reported in bohr, *a*_0_.

**Figure 4:**
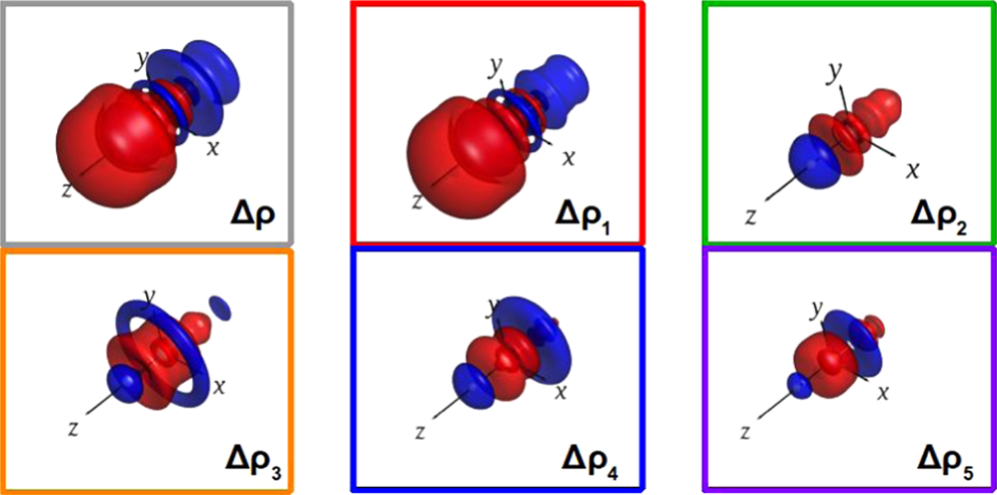
Isodensity surfaces of the total (Δρ) and of its first five NOCV components for the RgH_2_^–^ complex. The isovalue for the upper three surfaces (i.e., Δρ, Δρ_1_, Δρ_2_) is ±0.001 *e*/*a*_0_^3^, whereas for the three lower surfaces (i.e., Δρ_3_, Δρ_4_, Δρ_5_) is ±0.0001 *e*/*a*_0_^3^. Blue regions indicate electron density accumulation areas, and red regions indicate depletion areas.

**Figure 5:**
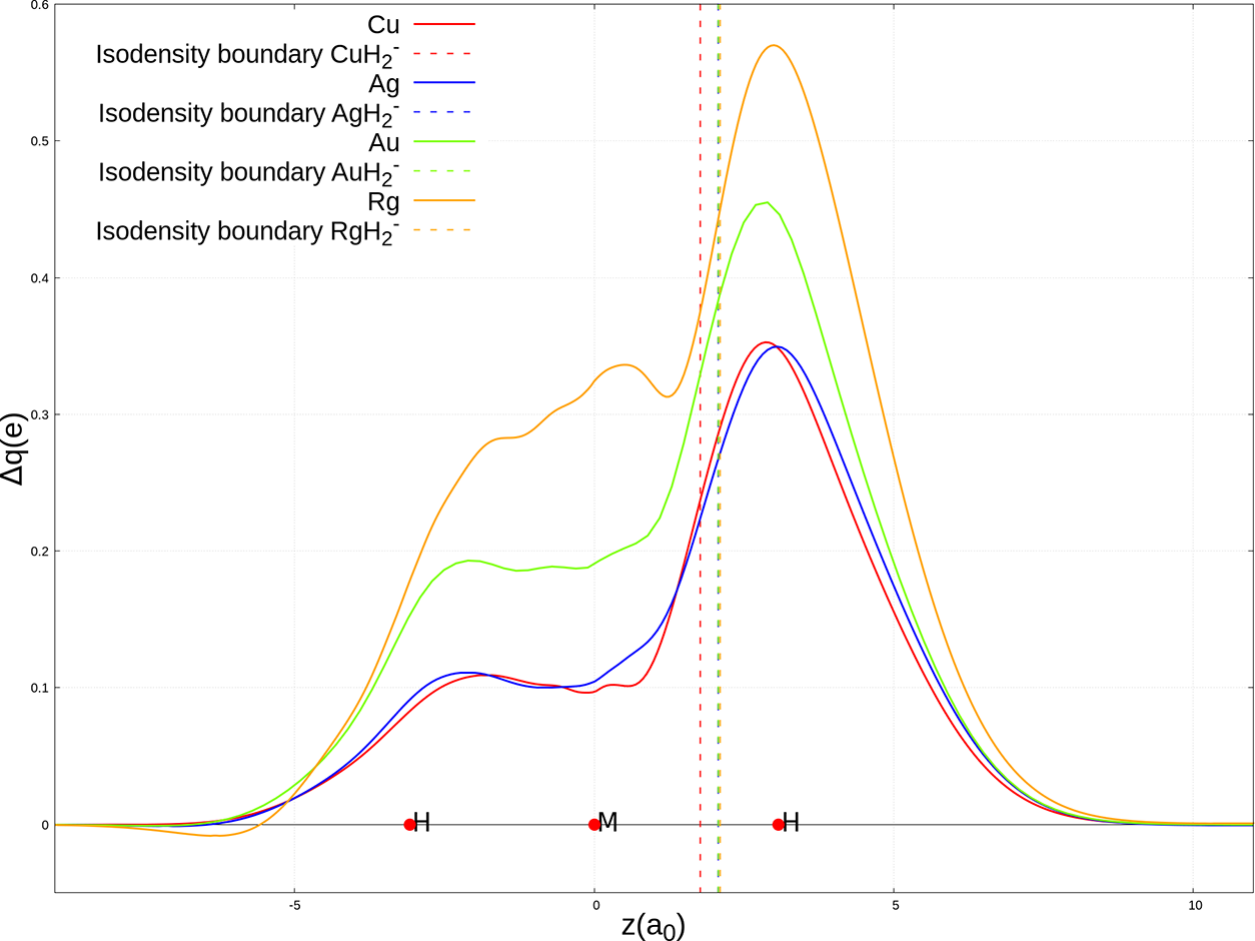
Charge displacement (CD) curves corresponding to the first NOCV (Δρ_1_) for all of the MH_2_^–^ complexes, M = Cu, Ag, Au, Rg. Red dots indicate the average position of the nuclei along the *z*-axis. Vertical dashed lines indicate the isodensity boundary for each complex. Distances are reported in bohr, *a*_0_.

Numerical results of the CD-NOCV analysis of the M–H bond in group 11 dihydrides are reported in Table 4. Since all of the four complexes show very similar bonding patterns, we show here the CD curves and isodensity pictures for RgH_2_^–^ (which could be considered as the most peculiar case) in Figures 3 and 4, respectively. The CD-NOCV analysis for copper, silver, and gold dihydrides is reported in the SI (Figures S6–S8). We compare the curves associated with the first NOCV (Δρ_1_) for all of the complexes in Figure 5. In addition, scalar relativistic CD-NOCV (“SR-CD”) analysis has also been carried out. The results of the SR-CD analysis are reported in Figures S9–S12 and in Table S5 in the SI.

**Table 4:** Charge Transfer (CT) and Eigenvalues (ν*_k_*) Corresponding to NOCVs for MH_2_^–^ Complexes^a^

	CuH_2_^–^		AgH_2_^–^		AuH_2_^–^		RgH_2_^–^	
*k*	CT*_k_* (*e*)	ν*_k_*	CT*_k_* (*e*)	ν*_k_*	CT*_k_* (*e*)	ν*_k_*	CT*_k_* (*e*)	ν*_k_*
net	0.214		0.234		0.328		0.408	
1	0.241	0.329	0.272	0.301	0.379	0.388	0.467	0.554
2	–0.021	0.107	–0.019	0.103	–0.035	0.108	–0.045	0.127
3	0.001	0.092	–0.011	0.094	0.001	0.081	0.001	0.049
4	–0.004	0.023	–0.004	0.022	–0.009	0.025	–0.010	0.038
5	–0.004	0.023	–0.004	0.022	–0.008	0.024	–0.004	0.036

a Overall net charge transfer values (entry labeled as “net”) for each complex are also reported.

The main picture emerging from our results is in close agreement with the previously depicted bonding schemes for MH_2_^–^ complexes.^30−33^ For each complex, the dominant component of the M–H bond (Δρ_1_, the red curve in the CD panels, Figure 3 and curves in Figure 5) is a CT from the H^–^ ligand toward the metal fragment, as it can be seen by the isodensity pictures (Figure 4), where a (red) electron density depletion area is present on the hydride fragment and a (blue) accumulation region is on the HM fragment. This component can be envisaged within the DCD model as a σ donation. Quantitatively, it widely varies along group 11: this component is very similar in magnitude for Cu and Ag (0.241 and 0.272 *e*, respectively) and the corresponding curves practically overlap (as shown in Figure 5). The CT values associated with this component increase sharply for Au (0.379 *e*) and Rg (0.467 *e*), showing therefore a great variability range along the group. The same trend (and very similar accumulation/depletion patterns) is observed for the overall net CT (associated with Δρ) that ranges from 0.214 to 0.408 *e*. The overall net CT variation is therefore mainly due to the σ donation bond component.

**Figure 6:**
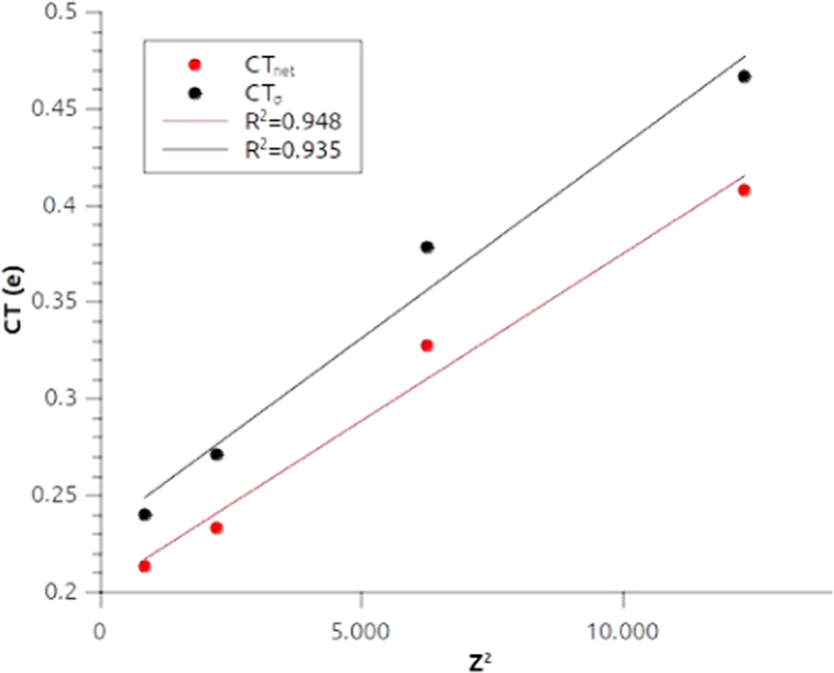
Correlation between the square of the atomic number (*Z*^2^) of the group 11 metal (M) and the net charge transfer (CT_net_) and the charge transfer associated with the first NOCV (CT_σ_) for the M–H bond in the MH_2_^–^ complexes series.

Figure 6 shows a very good agreement between the square of the atomic number of the metal and the CT values for both net and σ-donation charge transfer values. This correlation can be seen as an indirect relativistic effect: the increase of both the σ donation and the net CT, which is consistent with the increase of *Z*^2^ along the group, can be explained by the progressive relativistic stabilization of outer shell s orbitals on descending along group 11. This feature was also highlighted in ref (33) where it was held responsible for the increased covalency observed in these complexes. Indeed, we can interpret an increased σ donation from the hydride to the metal as an increased covalent character of the M–H bond.

This model becomes helpful when trying to rationalize the very high dissociation energies of AuH_2_^–^ and RgH_2_^–^. It has been shown that, in general, when a highly covalent bond is involved in the dissociation (for instance, in the dissociation of Au_2_), relativistic effects always tend to increase the dissociation energy because of the relativistic decrease of the overlap-related kinetic energy in the potential energy curve “well”.^93^ This phenomenon (i.e., the increased mass–velocity effect, which tends to lessen the overlap-related kinetic energy) can also be held responsible for the stronger M–H bond contractions in the Au and Rg cases. This explains the high *D*_e_ values for AuH_2_^–^ and RgH_2_^–^: the 6s and 7s orbitals, respectively, are so heavily stabilized by relativity that the overall M–H bond covalency increases, causing the dissociation energies to be highly increased by relativity.

A second bonding feature that also matches with the previously depicted bonding scheme is the unusual π back-donation from the metal fragment that populates the unoccupied high-lying 2p orbitals of the H^–^ fragment. The SR-CD analysis isodensity pictures (Figures S9–S12 in the SI) clearly show that NOCVs 4 and 5 result from the interaction of the outer d*_xz_* and d*_yz_* orbitals of the metal and the empty 2p atomic orbitals of hydrogen. Identifying at first glance this bond component in the framework of the CD-NOCV analysis based on the relativistic four-component framework is much less straightforward, due to the spinors’ mixing with symmetry. However, through a closer inspection, we can realize that NOCVs 4 and 5 can actually be related to the back-donation components by looking at the eigenvalues’ evolution along group 11. These NOCVs are degenerate for Cu (CT_4_ −0.004/CT_5_ −0.004) and Ag (CT_4_ −0.004/CT_5_ −0.004), and they become different for Au (CT_4_ −0.009/CT_5_ −0.008) and Rg (CT_4_ −0.010/CT_5_ −0.004), where spin–orbit coupling splits the two d orbitals involved in the π back-donation. Interestingly, the spin–orbit coupling is also responsible for changing the expected trend. From the SR-CD results, we see that the overall π back-donation increases from Cu to Rg (see CT values in Table S5 in the SI). However, if we look at the CT values reported in Table 4, we observe an increase from Cu to Au, but for RgH_2_^–^ one of the two components results to be quenched (CT_5_ = −0.004 *e*) and the overall π back-donation decreases. This finding is consistent with the fact that one of the two d orbitals gets heavily stabilized and therefore its interaction with the high-lying 2p orbitals of H^–^ is less effective. Although the overall effect of this back-donation component is expected to be small, this picture provides an interesting example where a four-component approach, which appropriately includes spin–orbit coupling in the calculations, is important to single out even the most subtle features of the chemical bond involving heavy and superheavy elements.

For the remaining NOCV components (i.e., *k* = 2 and 3), the Δρ_3_ component is not of particular interest for describing the M–H bond, since it involves charge accumulation and depletion on the *xy* plane, mainly on the metal, whereas the bond is oriented along the *z*-axis. The Δρ_2_ component, instead, shows charge flowing from the metal fragment toward the hydride ligand. Since both SR-CD and DKS-CD results highlight that this charge displacement has a cylindrical symmetry (σ), it can be interpreted as a σ back-donation. The original DCD scheme does not account for this component, and in this framework, it may actually have an artificial nature (due to the reference system adopted within the NOCV scheme) and may not be chemically significant. However, the capability of coinage metals of transferring electron charge from the metal toward the ligand via σ back-donation has been previously reported^94−96^ and it seems reasonable that this component could also be observed in this class of complexes.

### Mechanism of sd Hybridization in MH_2_^–^ Complexes

In the previous section, we have quantitatively described the main features of the M–H bond, its DCD bonding components, and their variation along the group, where the role of relativistic effects becomes crucial for AuH_2_^–^ and RgH_2_^–^. Nevertheless, we have not taken into account the role of sd hybridization in these complexes yet.

In general, the importance of sd hybridization in transition-metal (and in particular in coinage metal) complexes has already been well recognized.^97,98^ In particular, for gold, relativistic effects play a fundamental role in determining the extent of this hybridization. Indeed, the relativistic stabilization of the 6s orbital in Au makes it more prone to mix with 5d orbitals and, as a consequence, gold molecular compounds show peculiar features, such as the higher tendency of Au to form planar structure clusters with respect to Cu and Ag^99^ and the tendency of gold in its +1 oxidation state to form linear complexes with short Au(I)–L distances. The role of sd hybridization in determining such peculiar structures for Au(I) complexes has been quantitatively described in the case of the AuCN complex.^91,100^ It has been demonstrated that the M–C bond length in this complex is highly affected by the mechanism of sd hybridization. By picturing the sd hybridization as the removal of charge from the internuclear axis (i.e., where the d_*z*^2^_ orbital lies) to populate the outer shell s orbital, it is easy to see that on this axis, and one may expect that the repulsive barrier also weakens symmetrically, which would result in a tendency to have shorter bond distances and to form linear structures.

Previous studies report that bonding in group 11 dihydrides features the participation of sd hybrid orbitals, especially in AuH_2_^–^ and RgH_2_^–^, ^33^ and in this case we need to consider that sd hybridization may play a pivotal role also in determining the observed patterns for the spectroscopic constants. The effect of sd hybridization on group 11 compounds has also been discussed for explaining the bonding patterns observed for group 11 dimers, where it is expected to play a major role.^90^ In this last work, however, the role of sd hybridization has not been discussed from a quantitative perspective. The authors argued that the hybridization should be present to a greater extent in AuH_2_^–^ and RgH_2_^–^, favoring an increasingly covalent bond. However, despite its potential importance in determining and rationalizing structures, a quantitative assessment of the degree of hybridization (how many electrons are expected to be involved) is very scarce even in simple systems. For this reason, it would be very interesting to try to establish a more stringent link between the trends observed for bond lengths, dissociation energies, and force constants and the role of sd_*z*^2^_ hybridization in these complexes.

Recently, some of us provided a methodological advance for discussing quantitatively the effect of the sd hybridization in coinage metal cyanides. Indeed, for MCN complexes (M = coinage metals), which bear no trans ligand, the sd hybridization mechanism was analyzed in detail through the CD-NOCV analysis based on DKS calculations, which allowed a straight and unequivocal characterization of the charge rearrangement due to the sd hybridization in those complexes.^91^

Here, the presence of a trans ligand complicates the patterns of the charge rearrangements upon the formation of the HM···H^–^ bond and we are not able to get a clear picture of the sd hybridization mechanism occurring at the metal site. However, the selection of different starting fragments may be very helpful. Since the charge rearrangement takes place on the metal, we chose to perform the CD-NOCV analysis considering the M^+^ and H^–^···H^–^ fragments and inspecting different NOCVs to find proof for sd hybridization. We mention that in all cases we start from a nd^10^ configuration of the metal cation, which is the most natural choice for the comparative study we present in the following.

**Figure 7:**
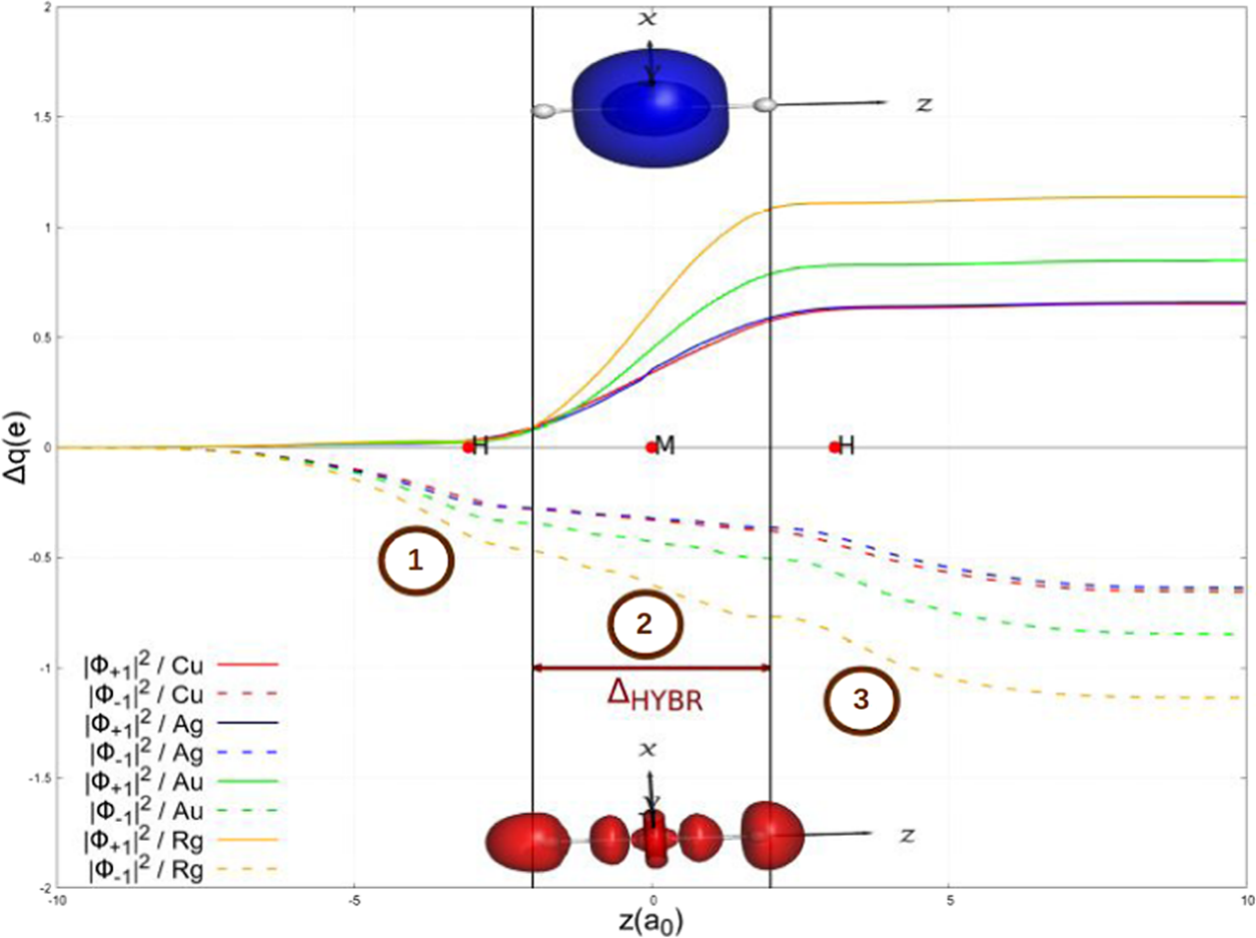
Comparison of charge-displacement (CD) curves for the MH_2_^–^ complexes, M = Cu, Ag, Au, Rg. The curves labeled as |Φ_–1_|^2^ (dashed lines) and |Φ_+1_|^2^ (solid lines) are scaled for the corresponding NOCV eigenvalue (ν_1_). The vertical black lines mark the average position of the isodensity boundaries for each complex and the average position of the H^–^ ligands on the *z*-axis (distance is in bohr, *a*_0_). The isodensity pictures (isovalue: ±0.005 *e*/*a*_0_^3^) corresponding to the two parent NOCV pair densities |Φ_–1_|^2^ and |Φ_+1_|^2^ have been shown only for the RgH_2_^–^ complex (similar shaped patterns are found for all MH_2_^–^ complexes), where red and blue regions correspond to electron charge depletion and accumulation areas, respectively.

The results are reported in Figure 7 and Table 5 and Figures S13–S16 in the Supporting Information.

**Table 5:** NOCV Eigenvalues for *k* = 1 (ν_1_) and Electronic Charge Associated with sd Hybridization (Δ_HYBR_) for the MH_2_^–^ Complexes, M = Cu, Ag, Au, Rg

group 11 dihydride	ν_1_	Δ_HYBR_ (*e*)
Cu	0.6540	0.0901
Ag	0.6352	0.0899
Au	0.8462	0.1607
Rg	1.1382	0.3026

The interpretation of the results is straightforward and very instructive. By inspection of the NOCVs and the corresponding parent densities |ϕ_±*k*_|^2^ (see Figures 7 and S13–S16), we observe that for Δρ_1_ (i.e., σ donation), the corresponding parent densities seem to represent the rearrangement that takes place after the sd hybridization. Indeed, by looking at the isodensity pictures, the |ϕ_–1_|^2^ density corresponds to a charge depletion on both the hydride ligands (due to the σ donation toward the metal cation) and the metal, where the depletion of electrons clearly resembles the shape of an atomic *n*d*_z_*^_2_^ orbital. For the |ϕ_+1_|^2^ density, we observe an electron density accumulation whose shape clearly recalls the (*n* + 1)s orbital and that takes place exclusively at the metal cation. Thus, the charge rearrangement can be regarded as the fingerprint of the sd hybridization mechanism, where electrons are moved from the d_*z*^2^_ orbital and populate the outer shell s orbital.

For a quantitative evaluation, we need to inspect the shape of the CD curves applied separately to the NOCV pair densities. The |ϕ_+1_|^2^ curve (solid lines in Figure 7) is flat in the area of the hydride ligands and therefore accumulation occurs only on the metal, in the region between the two isodensity boundaries. However, electrons that are accumulated in this region come from both σ donation and sd hybridization and therefore it would be hard to quantify the two contributions separately. The |ϕ_–1_|^2^ curve (dashed lines in Figure 7) is the key for quantifying the number of electrons involved in the sd hybridization. This curve must be read from the left to the right, and it can be divided (arbitrarily) into three different regions, separated by the two isodensity regions where the curve is flat (and therefore in that region, no rearrangement takes place). We observe the first depletion from the hydride ligand (region 1 in Figure 7) and then, in the area between the boundaries (region 2), which corresponds to the metal, we observe another depletion, where, however, the slope of the curve seems to be slightly different with respect to that in region 1 and corresponds to depletion of electrons from the d_*z*^2^_ orbital because of the sd hybridization rearrangement. Finally, a depletion on the other hydride ligand due to the σ donation (region 3) is observed. To quantify the electrons involved in sd hybridization, we have to analyze the region located between the two boundaries (i.e., region 2). Qualitatively, an inspection of the CD curves in this region (Figure 7) already gives an idea of the extent of the hybridization in these complexes. We see that in the interboundary region, the curves for Cu and Ag are very flat and they almost overlap. On going down the group 11, a greater slope for the curve of AuH_2_^–^ and an even greater slope for RgH_2_^–^ can be observed.

Note that, as mentioned above, the boundaries are located in nearly flat regions of the curve, thus confirming that this portion can be unambiguously separated.

We can evaluate the amount of electronic charge that is transferred from the d_*z*^2^_-shaped portion (region 2) of |ϕ_–1_|^2^ to |ϕ_+1_|^2^ (to which we refer to as “Δ_HYBR_”) by subtracting the values that the CD function associated with |ϕ_–1_|^2^ assumes at the boundaries. With this approach, we are able to exclude the electrons that are transferred to the metal by σ donation and only to account for the electrons associated with the sd hybridization mechanism. The data reported in Table 5 give a definitive and quantitative establishment of the relative importance of sd hybridization in the group 11 metals. Cu and Ag have practically the same small value of Δ_HYBR_ (less than 0.1 *e*). The value of Δ_HYBR_ for AuH_2_^–^ is almost twice larger than for the lighter coinage metals (almost 0.2 *e*). Concerning RgH_2_^–^, the amount of electrons involved in the sd hybridization mechanism is twice as bigger as gold’s and more than 3 times as bigger as Cu and Ag (more than 0.3 *e*).

### Spectroscopic Properties of MH_2_^–^ Complexes: Role of CT and sd Hybridization

In the above sections, we have clearly shown that, on descending along the periodic table (and thus on increasing the relativistic effects), the M–H bond increases its covalent character and the sd hybridization mechanism, occurring at the metal center, becomes more and more important. Both these effects are expected to play a key role in the reinforcement of the M–H bond on descending along the periodic table, and they may be used to rationalize the observed spectroscopic properties. As mentioned, the sd hybridization, in particular, is known to play a role in determining the shape and size of the metal by removing electron density from the bond axis and inducing a significant flattening at the metal, which may have a direct influence on the bond distances. Moreover, we can consider that both CT and sd hybridization are highly affected by relativistic effects, which stabilize, in particular, the *n*s atomic orbital of Au and Rg, thus favoring the mixing between s and d orbitals.

**Figure 8:**
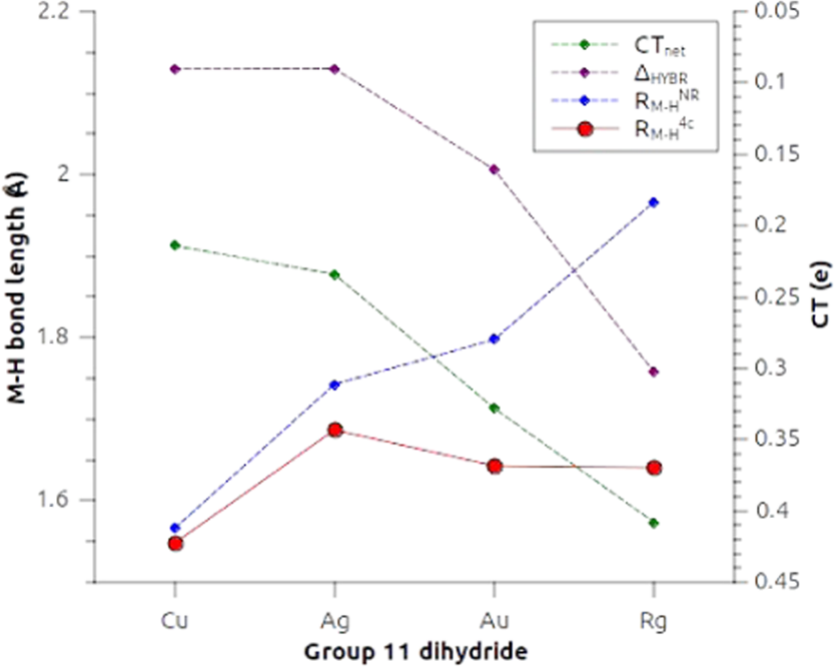
Nonrelativistic (*R*_M–H_^NR^, blue dashed line) and four-component (*R*_M–H_^4c^, solid red line) CCSD(T) M–H bond lengths (left axis) and CT_net_ (green dashed line) and Δ_HYBR_ (purple dashed line) values (right axis) for group 11 dihydrides.

In Figure 8, we try to establish a chemical model able to rationalize the trend observed for the M–H bond lengths (and for force constants and dissociation energies) by relating this trend with the calculated CT_net_ and Δ_HYBR_ values. In a nonrelativistic framework, where we expect that the extents of CT and sd hybridization are similar for all of the complexes, the bond lengths have a monotonic upward trend, with the M–H distances constantly increasing from Cu to Rg (dashed blue line in Figure 8). We clearly see that the trend for M–H bond lengths is modified in a four-component relativistic framework and that an evident correlation with the extent of the CT and the sd hybridization can be found.

We observe that, since the degree of sd hybridization and CT (i.e., a measure of the degree of covalency) are quite small for Cu and Ag, the Cu–H and Ag–H bond lengths are not heavily affected by the inclusion of relativistic effects and the nonrelativistic trend is practically unchanged. However, for Au and Rg, the larger extent of CT_net_ and sd hybridization heavily modifies the trend, with a contraction of the Rg–H bond by almost 0.5 Å which can be ascribed to the very large value of CT_net_ and Δ_HYBR_. It is interesting to note that, despite a systematic shift, the pattern of CT_net_ and Δ_HYBR_ is very similar along the group 11 series, suggesting that they arise from a common origin. In particular, we find that both sd hybridization and CT, playing a major role in characterizing the periodic trend, are strictly related to the stability of the outer (*n* + 1)s orbital, which is known to be strongly affected by the relativistic effects. In particular, the relativistic stabilization of the (*n* + 1)s (and the destabilization of the *n*d) orbital increases the mixing between the s and d orbitals and therefore favors sd hybridization. In other words, relativistically stabilized (*n* + 1)s orbitals (and a destabilized *n*d orbital) should increase the extent of hybridization and, as a consequence, the population of the (*n* + 1)s orbital as well. This model can be verified using the PA (see the Methods and Computational Details section for details), which allows the population of the atomic orbitals in the molecule to be checked. We carried out the analysis both in the nonrelativistic and in the four-component relativistic framework for group 11 anion dihydrides, and the results (reported in Table S6 in the SI) show two very different trends. Indeed, in the nonrelativistic framework, the population of both outer (*n* + 1)s and outer *n*d orbitals does not vary remarkably along the group. On the other hand, in a four-component framework, we see a trend that matches that observed for sd hybridization: the population of 4s and 5s orbitals in CuH_2_^–^ and AgH_2_^–^, respectively, is very similar (1.15 and 1.11 *e*, respectively), whereas the population of the 6s orbital of Au is much larger (1.40 *e*) and the population of the 7s orbital of RgH_2_^–^ is even larger (1.68 *e*). The correlation between the (*n* + 1)s orbital population and the extent of sd hybridization is tight, as reported in Figure S17 in the SI.

**Figure 9:**
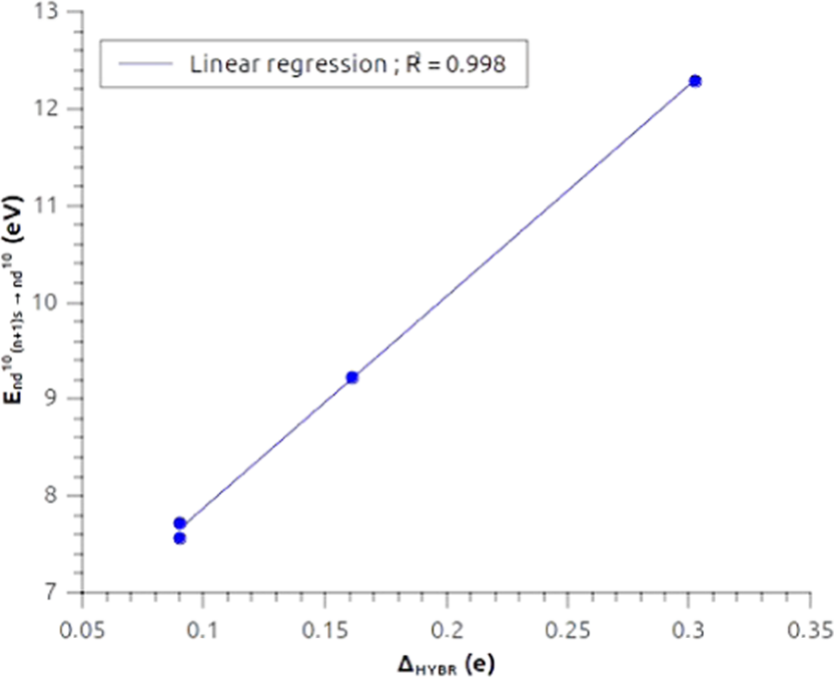
Correlation between calculated Δ_HYBR_ values for group 11 dihydrides and ionization energies for the *n*d^10^(*n* + 1)s → *n*d^10^ transition. For coinage metals, the experimental values from refs (101−103) have been used, whereas for Rg the four-component relativistic calculated energies from ref (104) have been used.

**Figure 10:**
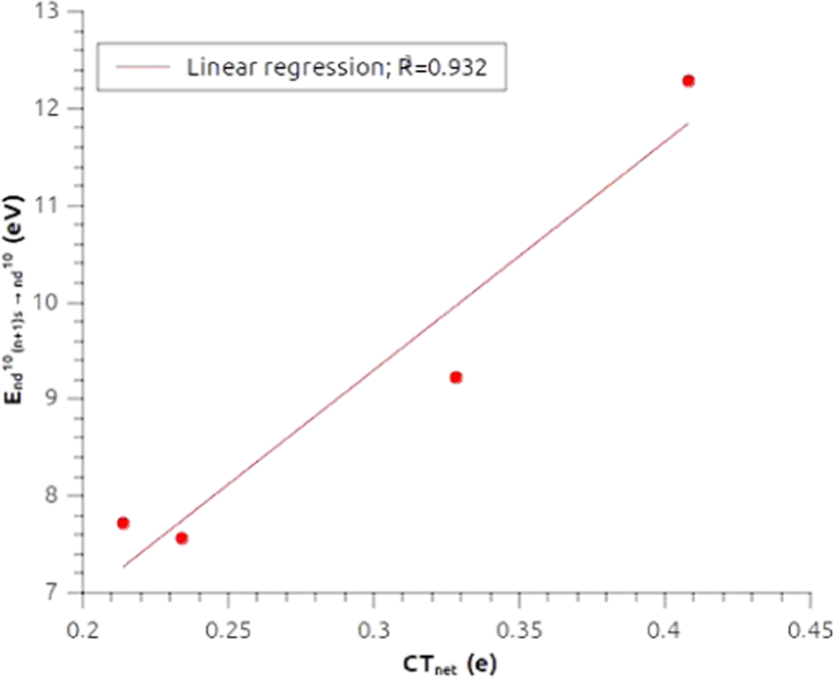
Correlation between calculated CT_net_ values for group 11 dihydrides and ionization energies for the *n*d^10^(*n* + 1)s → *n*d^10^ transition. For coinage metals, the experimental values from refs (101−103) have been used, whereas for Rg the four-component relativistic calculated energies from ref (104) have been used.

The relationship between sd hybridization and energy of the (*n* + 1)s orbital of the metal is shown in Figure 9, where we see a tight correlation between the calculated Δ_HYBR_ values and the energies associated with the *n*d^10^(*n* + 1)s → *n*d^10^ transition, which can be considered as a very clear measure of the energy of the (*n* + 1)s orbital in these species. Indeed, we observe that for Cu and Ag, where we find the outer shell s orbitals to be about at the same energy (since the relativistic stabilization of the latter should be very small), the extent of the hybridization is also small and practically equal, as demonstrated by the small and very similar values of Δ_HYBR_ reported. The picture dramatically changes for Au and Rg: here, the outer shell s orbitals are heavily stabilized, as demonstrated by the higher energies associated with the *n*d^10^(*n* + 1)s → *n*d^10^ transition. This causes the extent of hybridization to be higher (as demonstrated by the higher values of Δ_HYBR_). The energies of the relativistically stabilized outer shell (*n* + 1)s orbitals also correlate with the CT_net_ values, as shown in Figure 10, and this correlation demonstrates what we supposed before, i.e., that the observed trends for the net charge transfer (and the CT_σ_) can be ascribed to a heavy stabilization of the 6s and 7s orbitals of Au and Rg, respectively, causing the Au–H and Rg–H bonds to be very short and strong, with a high degree of covalency. We mention that analogous considerations can be applied to the observed trend for force constants and heterolytic dissociation energies: in that case, the highly covalent and short Au–H and Rg–H bonds display very high dissociation energies and force constants (and high stretching frequencies as well).

The picture we find here can also be understood from an elementary orbital interaction perspective. The (*n* + 1)s outer shell metal orbital mixes in with the *n*d_*z*^2^_ and the 1s orbital of hydrogen, in a typical case of a two-center-three-orbital interaction, yielding bonding, nonbonding, and antibonding σ molecular orbitals. This orbital interaction is responsible for electron charge depletion on both hydrogen 1s and metal *n*d_*z*^2^_ orbitals and electron charge accumulation on the metal (*n* + 1)s orbital: this is what we refer to as sd hybridization. On descending along group 11, the increasing stabilization of the (*n* + 1)s orbital toward the heavier Au and Rg metals by relativistic effects is key, since its contribution to the resulting molecular orbital increases as well as the amount of *n*d_*z*^2^_ → (*n* + 1)s CT (sd hybridization).

In conclusion, these findings, together with the previously reported results of the DKS-CD analysis, represent an original key for interpreting in terms of a chemical bond the periodic trends of the spectroscopic constants of group 11 dihydrides and, in general, heavy and superheavy metals’ compounds.

## Conclusions

A systematic analysis of the main spectroscopic constants (equilibrium bond lengths, heterolytic dissociation energies, and force constants) has been carried out for group 11 dihydrides (MH_2_^–^, M = Cu, Ag, Au, Rg). A highly accurate relativistic four-component CCSD(T) approach was used for calculating these quantities. Equilibrium M–H distances show a “V” shaped trend, with Ag having the longest distance (1.687 Å) and Au and Rg having very short M–H bond lengths (1.643 and 1.641 Å, respectively). Similarly to the well-studied monohydrides, it has been shown that the relativistic contraction of the M–H bond in dihydrides tightly correlates with the square of the atomic number of the metal. A very similar trend is displayed for heterolytic dissociation energies and force constants (and stretching frequencies), where we observe the lowest *D*_e_ and *k*_M–H_ values for AgH_2_^–^ (72.61 kcal/mol and 130.9 N/m, respectively) and the highest values for RgH_2_^–^ (98.94 kcal/mol and 214.6 N/m), which also shows the highest antisymmetric stretching frequency (2285.9 cm^–1^).

The relativistic four-component CD-NOCV analysis has been used for describing the M–H bond and rationalizing the observed trends for the spectroscopic constants. The M–H bond has been found to be mainly driven, as previously reported, by a strong ligand-to-metal σ donation that varies widely and monotonically along the series from Cu (0.241 *e*) to Rg (0.467 *e*). The progressive stabilization of the outer shell s orbitals on descending along group 11 favors an increased σ donation and an increased covalency of the M–H bond. The CD-NOCV approach has also been used to quantify the extent of the sd hybridization that occurs at the metal in these complexes by relying on an original approach, where different starting fragments for the analysis (i.e., M^+^ and H^–^···H^–^) have been used. Results show that, while the extent of hybridization is very small for Cu and Ag (less than 0.1 *e*), it becomes increasingly important for Au and Rg (0.16 and 0.30 *e*, respectively). It has been quantitatively demonstrated that the extent of sd hybridization (Δ_HYBR_) strictly depends on the relativistic stabilization of the outer shell s orbitals. Indeed, Δ_HYBR_ values tightly correlate with the energy involved in the removal of an electron from the outer shell s orbital, which can be considered as a measure of the energy of the *n*s orbitals.

The sd hybridization and CT trends are able to rationalize the “V”-shaped pattern observed for the bond lengths (and dissociation energies and force constants). The relativistically enhanced sd hybridization favors shorter metal–ligand distances by removing electron density from the bond axis and therefore the high degree of hybridization is consistent with the very short and strong Au–H and Rg–H bonds and, in general, with the peculiar “V”-shaped patterns observed for these complexes. This work represents an original contribution to the interpretation of the spectroscopic/bond property relationship in these understudied complexes, and it paves the way for using the four-component relativistic approach within a highly accurate framework for investigations of compounds containing heavy and superheavy elements.

## Supporting Information Available

Methodology and computational details, DFT calculations for the exchange–correlation functional and relativistic Hamiltonian testing, NR and 4c-CCSD(T) PESs, DKS CD-NOCV analysis for CuH_2_^–^, AgH_2_^–^, and AuH_2_^–^, SR-CD analysis and DKS-CD analysis using M^+^ and H^–^···H^–^ fragments, (*n* + 1)s and *n*d orbital populations, and correlation between the (*n* + 1)s orbitals population and sd hybridization extent (Δ_HYBR_) (PDF)
