# Supplementary material for: Spectroscopic/Bond Property Relationship in Group 11 Dihydrides via Relativistic Four-Component Methods
Source: J Phys Chem A. 2020 Dec 2;124(50):10565–79. doi: 10.1021/acs.jpca.0c09043 (PMC8016197; doi:10.1021/acs.jpca.0c09043)
Supplement: Supplementary file 1 — jp0c09043_si_001.pdf [file jp0c09043_si_001.pdf]

Supporting Information

Spectroscopic/Bond Property Relationship in Group 11 Dihydrides via Relativistic Four-Component Methods

Diego Sorbelli [a], Matteo De Santis [ab], Paola Belanzoni [abc*], Leonardo Belpassi [bc*]

[a] Department of Chemistry, Biology and Biotechnology, University of Perugia, via Elce di Sotto 8, 06123 Perugia, Italy

[b] CNR Institute of Chemical Science and Technologies "Giulio Natta" (CNR-SCITEC), via Elce di Sotto 8, 06123 Perugia, Italy

[c] Consortium for Computational Molecular and Materials Sciences (CMS)2, via Elce di Sotto 8, 06123 Perugia, Italy

Contents

Methodology and Computational Details S2
Tables S1-S2 .... S6
Table S3........ S7
Figure S1 .... S8
Table S4........ S9
Figure S2-S5: NR and 4c-CCSD(T) PESs for Group 11 dihdyrides S10
Figures S6-*S8* S14
Figures S9-*S12* S17
Table S5........ S21
Figures S13-S16........ S22
Table S6........ S24
Figure S17 .... S25
References........ S26

Methodology and Computational Details

Methodological details of the 4c-CCSD(T) geometry optimizations

Geometries have been optimized at the 4c-CCSD(T) level by performing the numerical first derivatives of the energy with respect to the nuclear coordinates, by considering several conditions: i) the geometries are expected to be linear from previous calculations[1–4]; ii) the two M-H bonds are considered to be equal, on the basis of the complexes linear centrosymmetric symmetry ($D_{\infty h}$). According to these conditions, we could optimize the MH_2^- complexes by only accounting for one degree of freedom (the M-H bond) in an iterative approach. At each step, three single-point calculations were performed and a second order Newton's interpolation procedure was used for obtaining the minimum energy and the corresponding bond length. The steps have been repeated until convergence between the two bond lengths obtained after two consecutive steps was reached (10^{-4} Å). Using this approach, it was possible to optimize the geometries with a computationally demanding state-of-the-art method in a reasonable amount of time.

Methodological details of the 4c-CCSD(T) vibrational frequencies calculations

The Hessian matrix has been calculated considering that, since in this class of linear triatomic centrosymmetric molecules the stretching and bending modes are not coupled, we could neglect any motion along the x and y axis and considering motion only along the z-axis. In this way, the Hessian is simplified and becomes a 3x3 matrix.

The elements of the Hessian have been calculated via numerical derivatives. The most convenient approach in our case is the central difference approximation. In this way first derivatives can be expressed as follows:

$$f'(z_0) = \frac{f(z_0+h)-f(z_0-h)}{2h} + \varepsilon h^2 \qquad (1)$$

where h is the displacement from the equilibrium x_0 value; $f(x_0+h)$ and $f(x_0-h)$ represent the function evaluated at both forward and backward displacements and εh^2 represents the error due to the approximation. This is a more convenient way of evaluating derivatives with respect to backward of forward difference approximation, since the error scales as h^2.

In our case, the diagonal elements of the Hessian can be simply expressed as:

$$k_{ii} = \frac{\delta^2 V}{\delta z_i^2} = \frac{f(z_0+h)-2f(z_0)+f(z_0-h)}{h^2} + \varepsilon h^2 \quad (2)$$

Instead, in the case of non-diagonal elements, we need to perform subsequent first derivatives for calculating the gradients (G_i^+ and G_i^-) at first and then the element of the Hessian matrix:

$$k_{ij} = \frac{\delta^2 V}{\delta z_i z_j} = \frac{\delta G_i}{\delta z_j} = \frac{G_i^+ - G_i^-}{2h_j} + \varepsilon h_j^2 \quad (3)$$

A critical choice for a good approximation is in the value of h. A too small value of h may cause problems in the sense that the difference between the function evaluated at backwards and forward displacement may be smaller than the precision of the calculations, therefore causing the final values to oscillate and therefore not being accurate. Instead, a too big value of h may cause problems concerning a too big error in the estimation of the derivative and therefore a bad approximation of the latter.

With the aim of understanding which the optimal value of h for introducing the smallest possible error was, we calculated the elements of the Hessian for CuH_2^- with different values of h ranging from 0.005 to 0.05. Then, rearranging Eqs. (2) and (3), we could find two expressions that, via linear or polynomial regression, could allow us to estimate the error we introduce in the approximation and therefore the real force constant.

For diagonal elements, rearranging Eq. (2) we obtain:

$$k_{ii}^{appr} = k_{ii}^{real} + \varepsilon_{ii} h^2 \quad (4)$$

This means that in both cases we should be able, by plotting the $k_{ii}^{apparent}$ values (which are the ones we determine through our calculations) versus h^2, by the means of a linear regression, we should be able to interpolate them to a straight line, whose intercept corresponds to the real force constant.

In the case of non-diagonal elements, manipulating the expressions for the gradients and the final derivative, we obtain the following expression:

$$k_{12}^{apparent} = k_{12}^{real} + \left(\frac{\varepsilon_{12}^{+} - \varepsilon_{12}^{+}}{2}\right) h + \varepsilon_{k12} h^2 \quad (5)$$

This means that for k_{12} (and k_{13} , for which one obtains the same equations), one can estimate the error due to the approximation by plotting the $k_{12}^{apparent}$ values versus h and interpolate the data with a second-order polynomial.

Below are shown the plots for the four constants and the k_{ij}^{real} values, comparing the results with the ones where the smallest value of h is used (i.e. 0.005 Å).

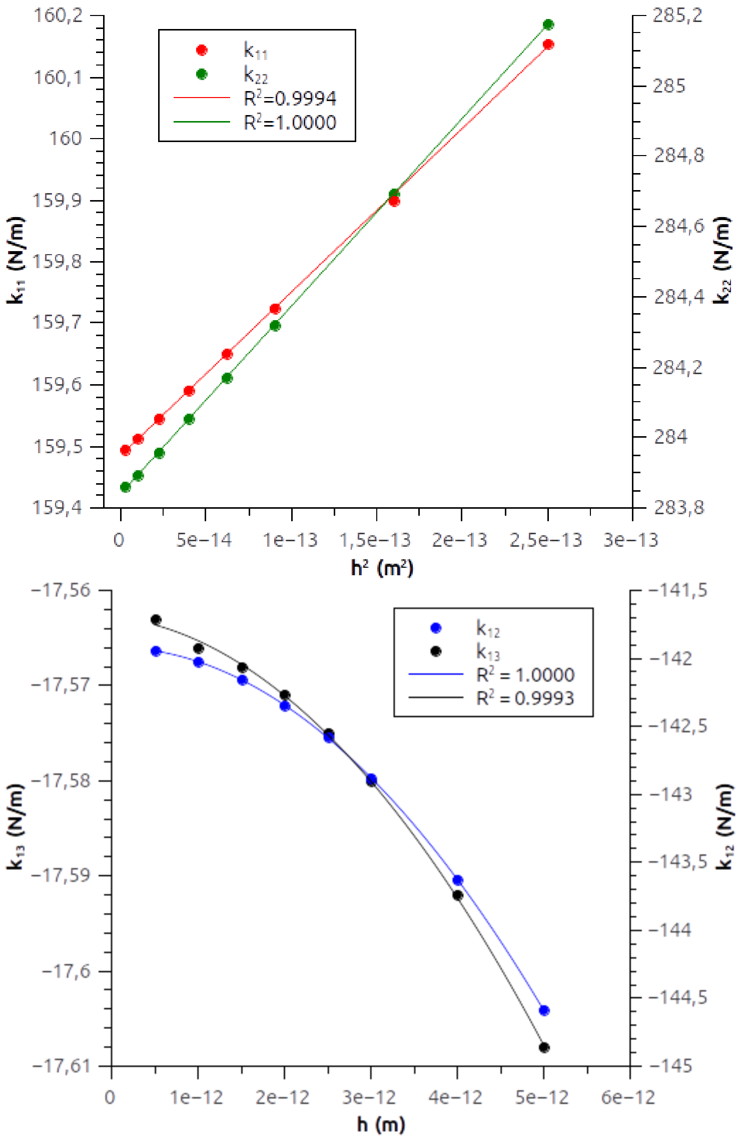

Figure M1. Plot of h^2 values versus the calculated k_{ii} elements (top panel) and h values versus calculated k_{ij} elements (bottom panel) for CuH_2^-. The calculations have been carried out with the 4c-CCSD(T) approach and a dyall.tvz basis set.

Force constant	k^{real} (N/m)	k with h=0.005 Å (N/m)
k_{11}	159.485 ± 0.002	159.495
k_{12}	-141.919 ± 0.002	-141.946
k_{22}	283.840 ± 0.001	283.859
k_{13}	-17.563 ± 0.001	-17.563

Table M1. Comparison between “real” extrapolated k values (k^{real}) and k values calculated awith h=0.005 Å for CuH_2^-.

The polynomial interpolations are very accurate (as shown by the R^2 values equal or extremely close to 1). We show how the k_{ij}^{real} values obtained are extremely close to the ones we report using a finite h value of 0.005 Å, leading to practically identical stretching frequencies. At this point, it is reasonable to believe that the central differences approximation works very well for estimating the stretching frequencies of CuH_2^-, with h=0.005 Å being an appropriate value of displacement to perform these calculations.

Computational details of the DKS-CD analysis

The CD analysis has also been applied to quantify the rearrangement of the electron density in the MH_2^- complexes at relativistic four-component Dirac-Kohn-Sham level[5] using BERTHA code[6–9] with the following computational set-up. The large component of the basis set for all the atoms was generated by uncontracting triple-ζ quality Dyall’s basis sets[10–13].The corresponding small component basis was generated using the restricted kinetic balance relation[14]. For the metal atoms (i.e. Cu, Ag, Au, Rg), a previously optimized auxiliary basis set for density fitting denoted as B20 was used[15]. For H atoms, accurate auxiliary basis sets were generated using a simple procedure starting from available DeMon[16] Coulomb fitting basis set. It is worth recalling that the Hermite Gaussian Type Functions (HGTFs) used as fitting functions are grouped together in sets sharing the same exponents (analogous scheme is adopted in the nonrelativistic DFT code DeMon)[16]. The sets are formed so that to an auxiliary function of a given angular momentum all the functions of smaller angular momentum are associated. Consequently, due to the variational nature of the density fitting procedure implemented, a fitting basis set of increased accuracy can be generated by simply upshifting the angular momentum in the basis set definition[17]. For H we achieved a fitting basis set of higher accuracy (referred to as A2) simply by up-shifting of two units the angular momentum of all the DeMon Coulomb Fitting definitions. This assures an accuracy on the Coulomb energy of 0.03 mHartree. The PBE[18] functional was used. An energy convergence criterion of 10^{-7} Hartree on the total energy was adopted.

Table S1. DFT SOC-ZORA optimized Au-H bond lengths in AuH_2^- by using different exchange-correlation functionals. CCSD(T)-SO calculated Au-H bong length from ref. 2 is used as reference.

Reference Au-H bond length: 1.647 Å			
**Functional**	**Au-H Bond length (Å)**	**Functional**	**Au-H Bond length (Å)**
BLYP	1.666	S12H	1.661
PBE	1.658	M06-L	1.664
BP86	1.657	TPSS	1.659
B3LYP	1.659	M06	1.680
PBE0	1.650	TPSSh	1.656

Table S2. DFT SR-ZORA optimized Au-H bond lengths in AuH_2^- by using different exchange-correlation functionals. CCSD(T)-SO calculated Au-H bong length from ref. 2 is used as reference.

Reference Au-H bond length: 1.647 Å			
**Functional**	**Au-H Bond length (Å)**	**Functional**	**Au-H Bond length (Å)**
BLYP	1.672	S12H	1.667
PBE	1.663	M06-L	1.670
BP86	1.662	TPSS	1.665
B3LYP	1.664	M06	1.687
PBE0	1.656	TPSSh	1.662

Table S3. 4-component relativistic (R^{4C}) and non relativistic (R^{NR}) calculated M-H equilibrium bond lengths for MH complexes. The Relativistic Bond Contraction (RBC) for each complex is reported. When available, the experimental value (R^{EXP}) from ref. 19 is reported for comparison.

**Group 11 hydride**	**R^{EXP} (Å)**	**R^{4C} (Å)**	**R^{NR} (Å)**	**RBC (pm)**
Cu	1.463	1.458	1.484	2.6
Ag	1.618	1.615	1.691	7.6
Au	1.524	1.521	1.740	21.9
Rg	/	1.521	1.925	40.4

Figure S1. Correlation between the square of the atomic number (Z^2) and the relativistic bond contraction (RBC) in MH complexes. R^2=0.998.

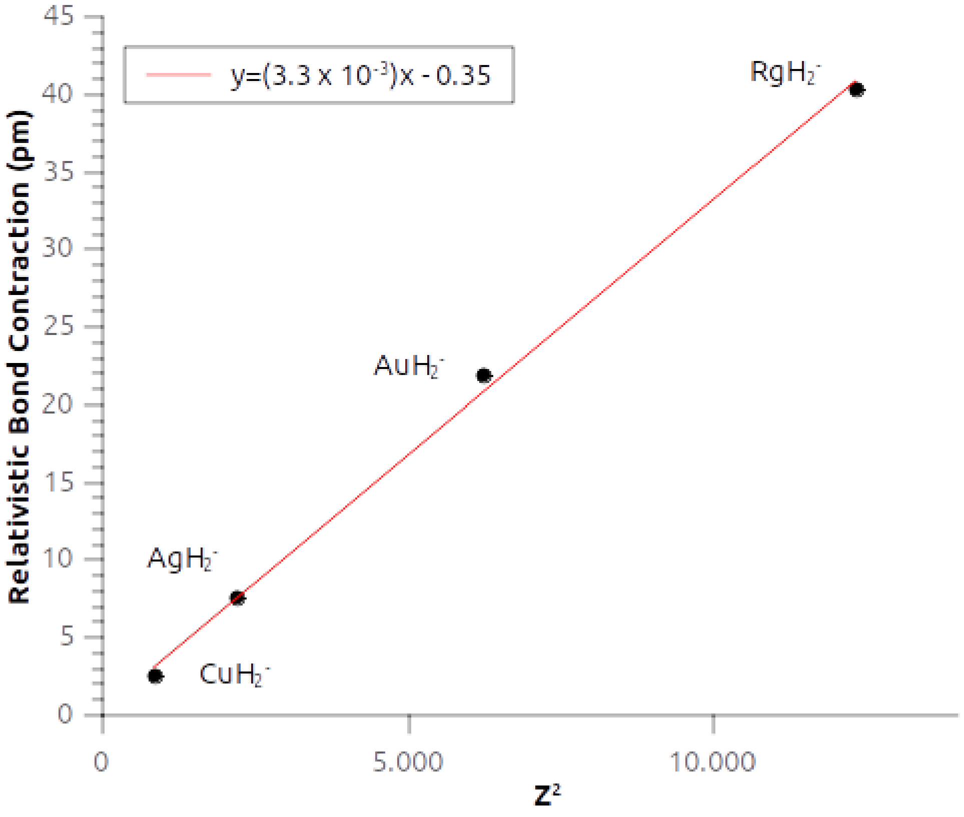

Table S4. DFT SR-ZORA (ΔE_{INT}^{SR}) and SOC-ZORA (ΔE_{INT}^{SOC}) calculated interaction energies for AuH_2^- by using different exchange-correlation functionals.

**Functional**	**ΔE_{INT}^{SR} (kcal/mol)**	**ΔE_{INT}^{SOC} (kcal/mol)**	**Functional**	**ΔE_{INT}^{SR} (kcal/mol)**	**ΔE_{INT}^{SOC} (kcal/mol)**
BLYP	90	83	S12H	87	85
PBE	89	87	M06-L	85	83
BP86	88	86	TPSS	89	87
B3LYP	86	84	M06	87	85
PBE0	90	88	TPSSh	89	87

Figure S2. Non relativistic and 4c-CCSD(T) calculated PESs for the HCu---H$^-$ bond. The energy has been shifted according to the two fragments being at infinite distance.

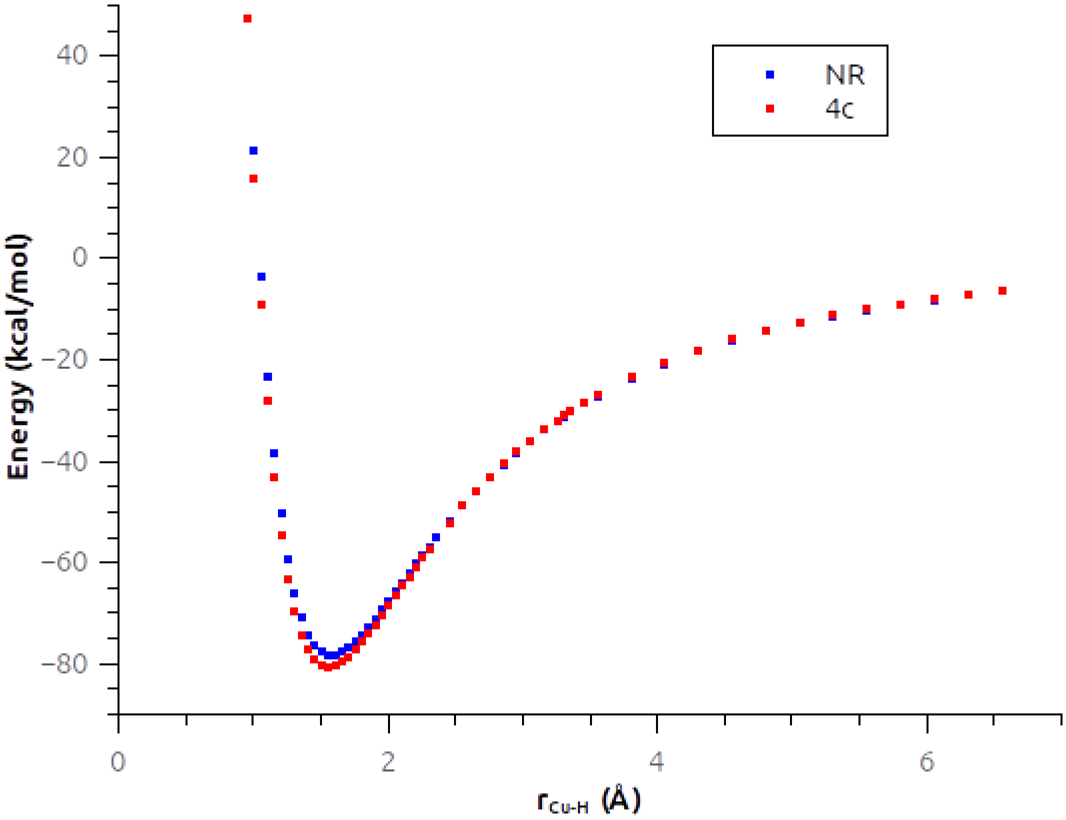

Figure S3. Non relativistic and 4c-CCSD(T) calculated PESs for the HAg---H$^-$ bond. The energy has been shifted according to the two fragments being at infinite distance.

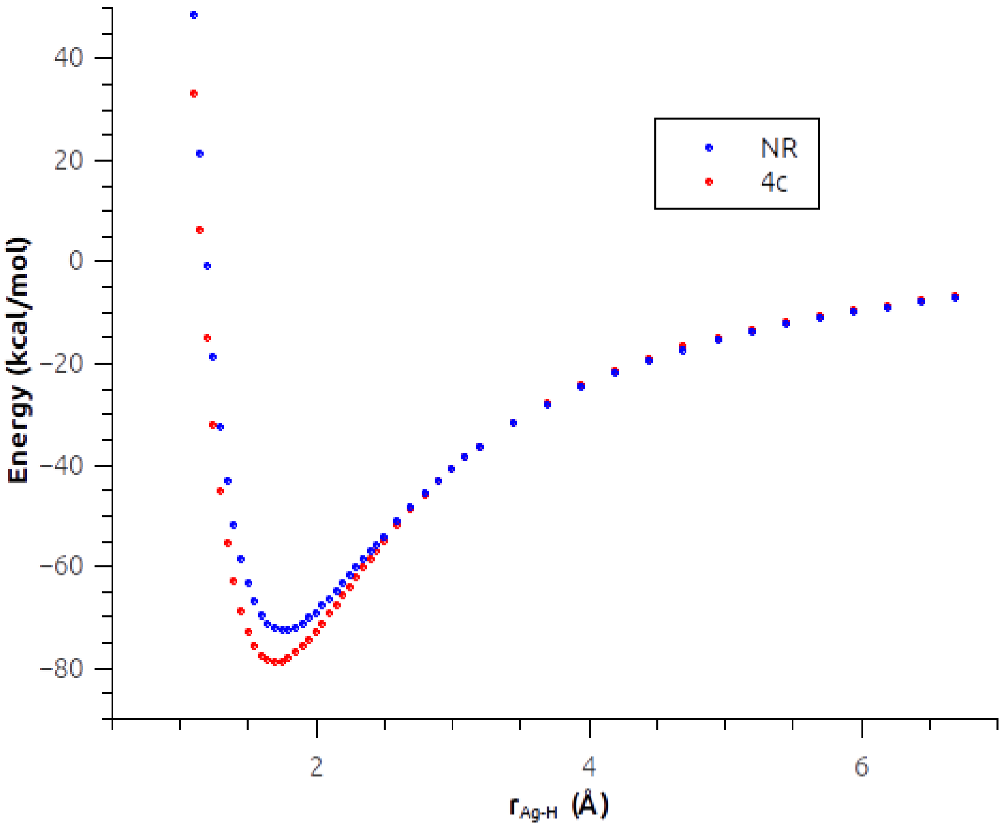

Figure S4. Non relativistic and 4c-CCSD(T) calculated PESs for the HAu---H^- bond. The energy has been shifted according to the two fragments being at infinite distance.

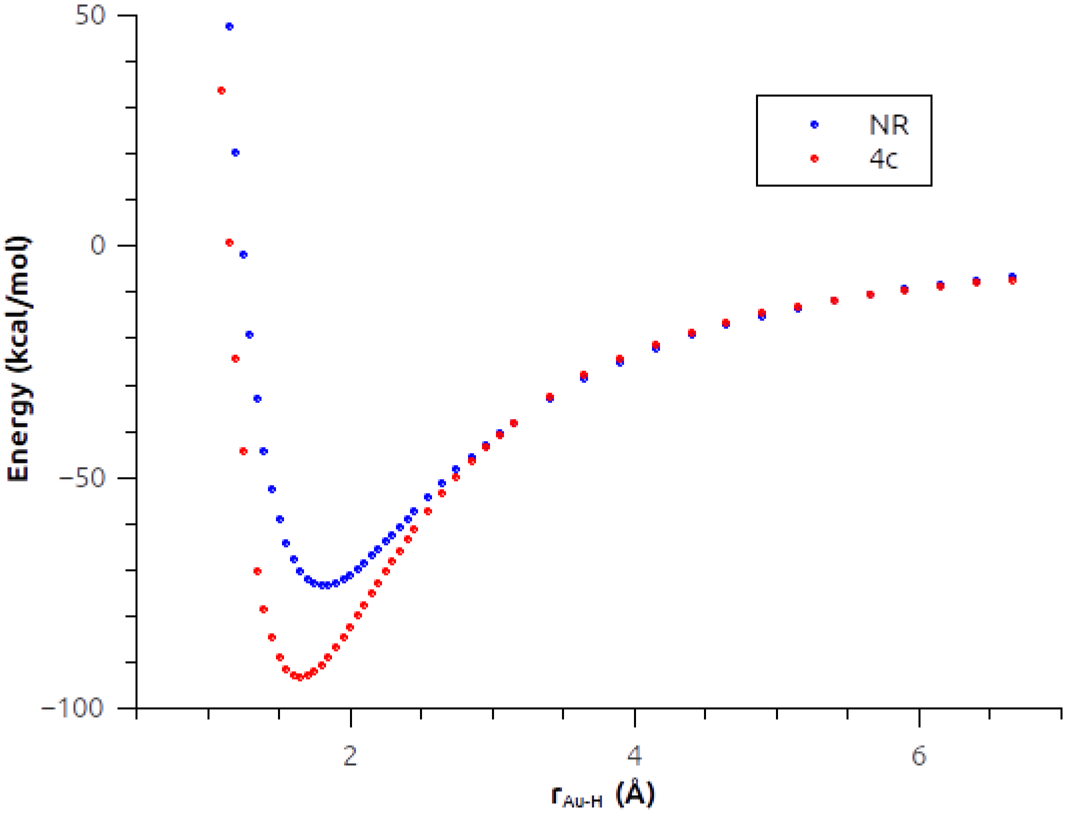

Figure S5. Non relativistic and 4c-CCSD(T) calculated PESs for the HRg---H^{-} bond. The energy has been shifted according to the two fragments being at infinite distance.

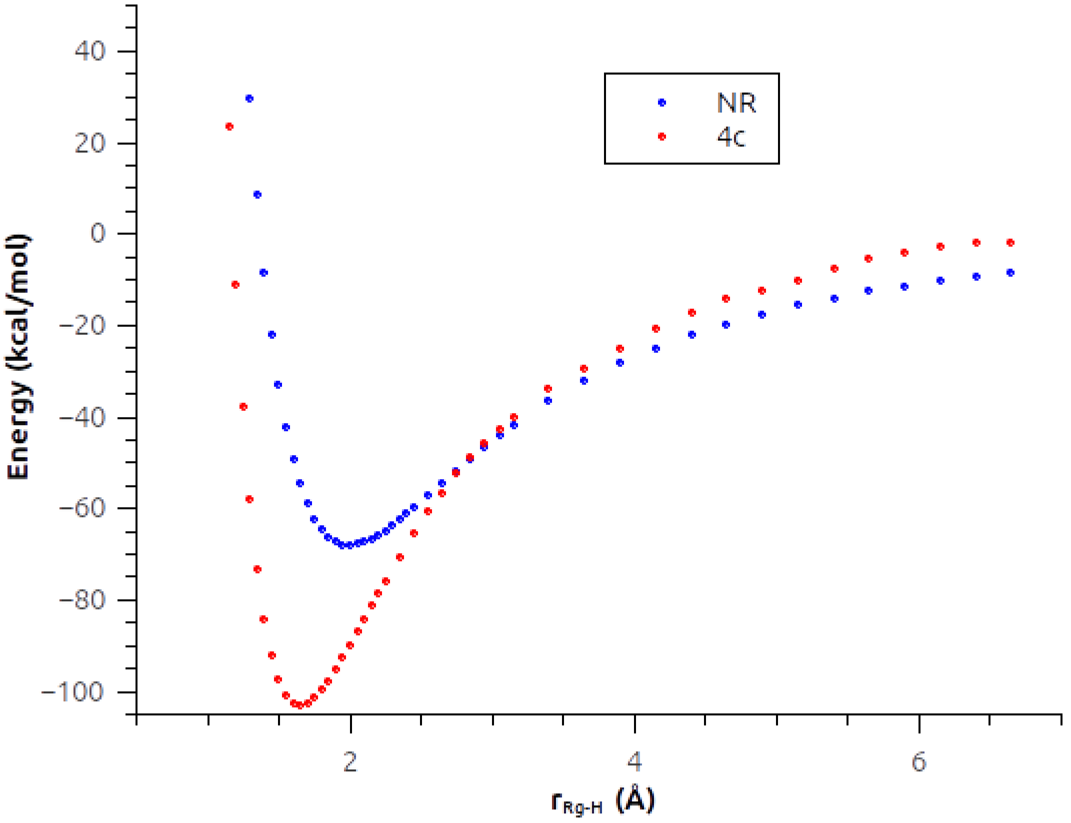

Figure S6. *Top:* DKS-CD calculated isodensity surfaces of the total (Δρ) and of its first five NOCV components for the CuH_2^- complex. The isovalue for the upper three surfaces (i.e. Δρ, $Δρ_1$, $Δρ_2$) is ±0.001 e a_0^{-3}, whereas for the three lower surfaces (i.e. $Δρ_3$, $Δρ_4$, $Δρ_5$) the isovalue is ±0.0001 e a_0^{-3}. Blue regions indicate electron charge accumulation areas, whereas red regions indicate depletion areas.

Bottom: corresponding DKS-CD curves for the CuH_2^- complex. Red dots indicate the position of the nuclei along the z axis. The vertical line marks the isodensity boundary.

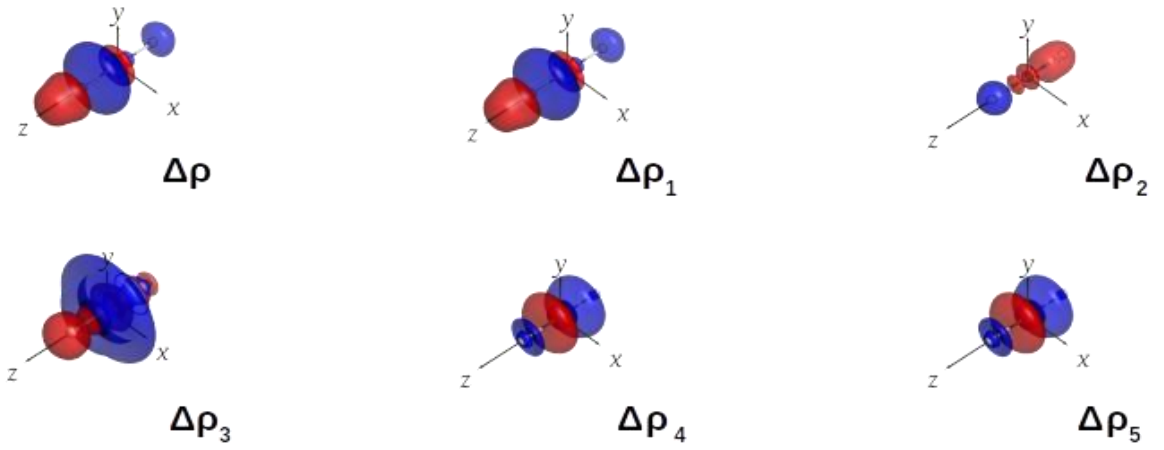

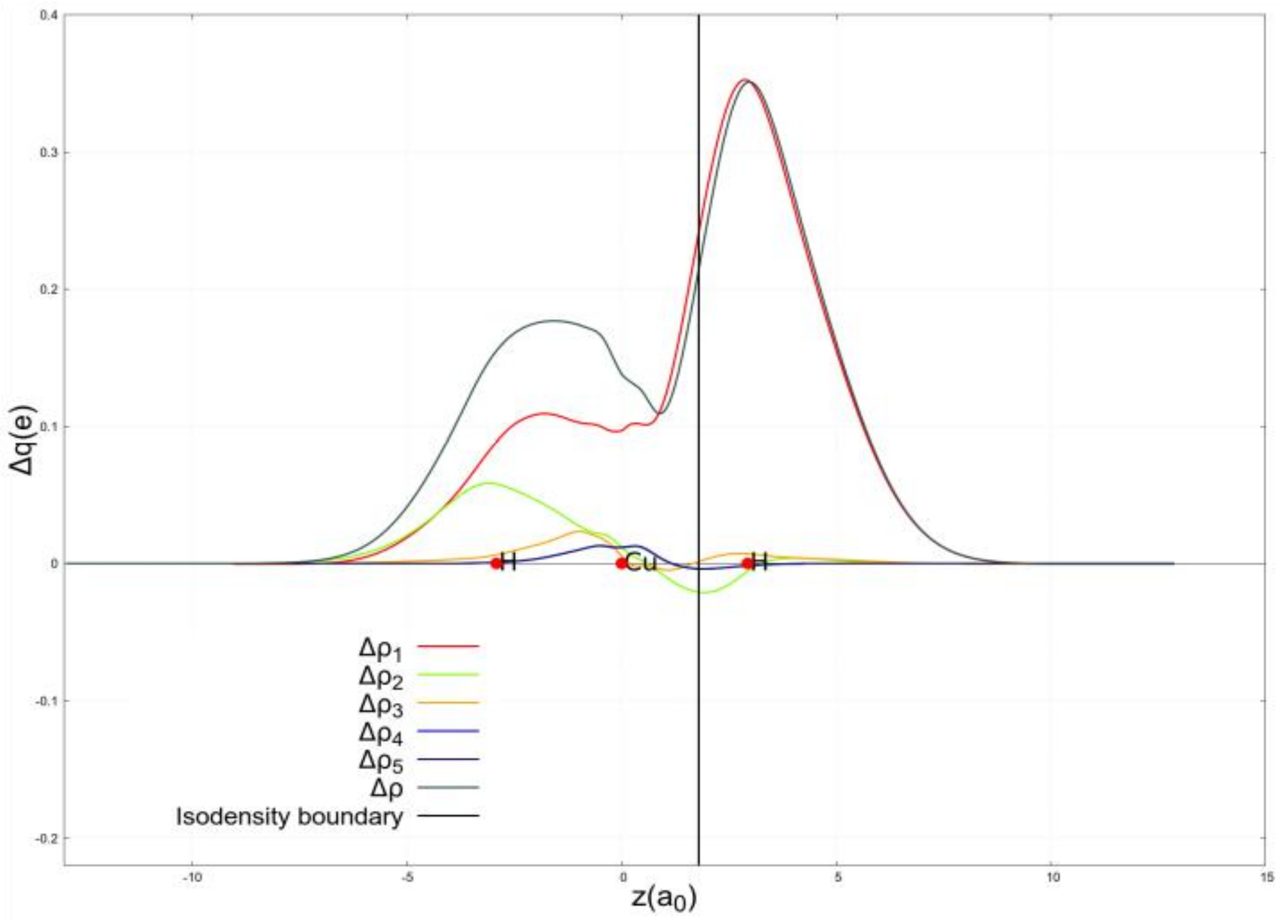

Figure S7. *Top:* DKS-CD calculated isodensity surfaces of the total (Δρ) and of its first five NOCV components for the AgH_2^- complex. The isovalue for the upper three surfaces (i.e. Δρ, $\Delta\rho_1$, $\Delta\rho_2$) is ±0.001 e a_0^{-3}, whereas for the three lower surfaces (i.e. $\Delta\rho_3$, $\Delta\rho_4$, $\Delta\rho_5$) the isovalue is ±0.0001 e a_0^{-3}. Blue regions indicate electron charge accumulation areas, whereas red regions indicate depletion areas.
Bottom: corresponding DKS-CD curves for the AgH_2^- complex. Red dots indicate the position of the nuclei along the z axis. The vertical line marks the isodensity boundary.

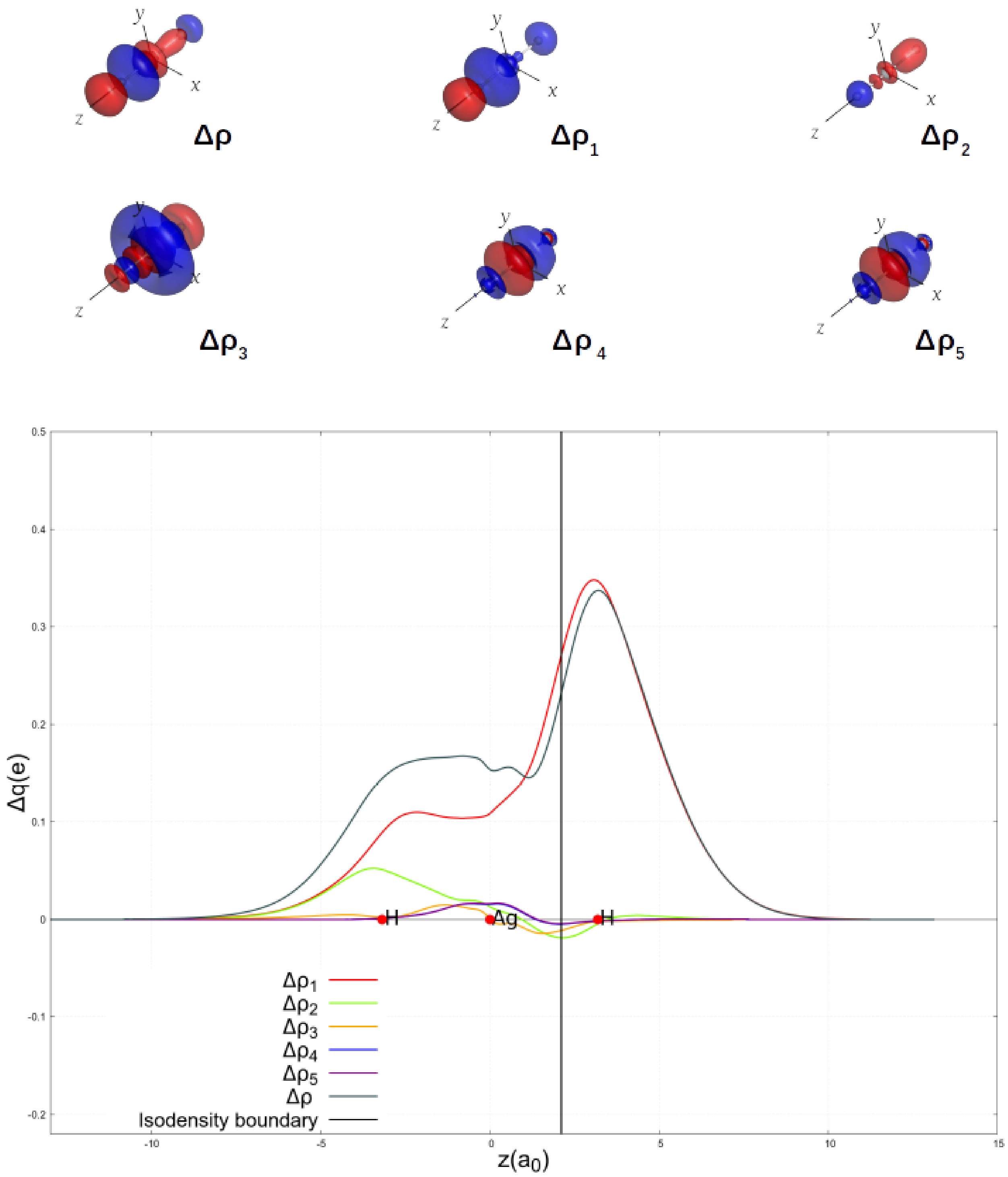

Figure S8. *Top:* DKS-CD calculated isodensity surfaces of the total (Δρ) and of its first five NOCV components for the AuH_2^- complex. The isovalue for the upper three surfaces (i.e. Δρ, $Δρ_1$, $Δρ_2$) is ±0.001 e a_0^{-3}, whereas for the three lower surfaces (i.e. $Δρ_3$, $Δρ_4$, $Δρ_5$) the isovalue is ±0.0001 e a_0^{-3}. Blue regions indicate electron charge accumulation areas, whereas red regions indicate depletion areas.

Bottom: corresponding DKS-CD curves for the AuH_2^- complex. Red dots indicate the position of the nuclei along the z axis. The vertical line marks the isodensity boundary.

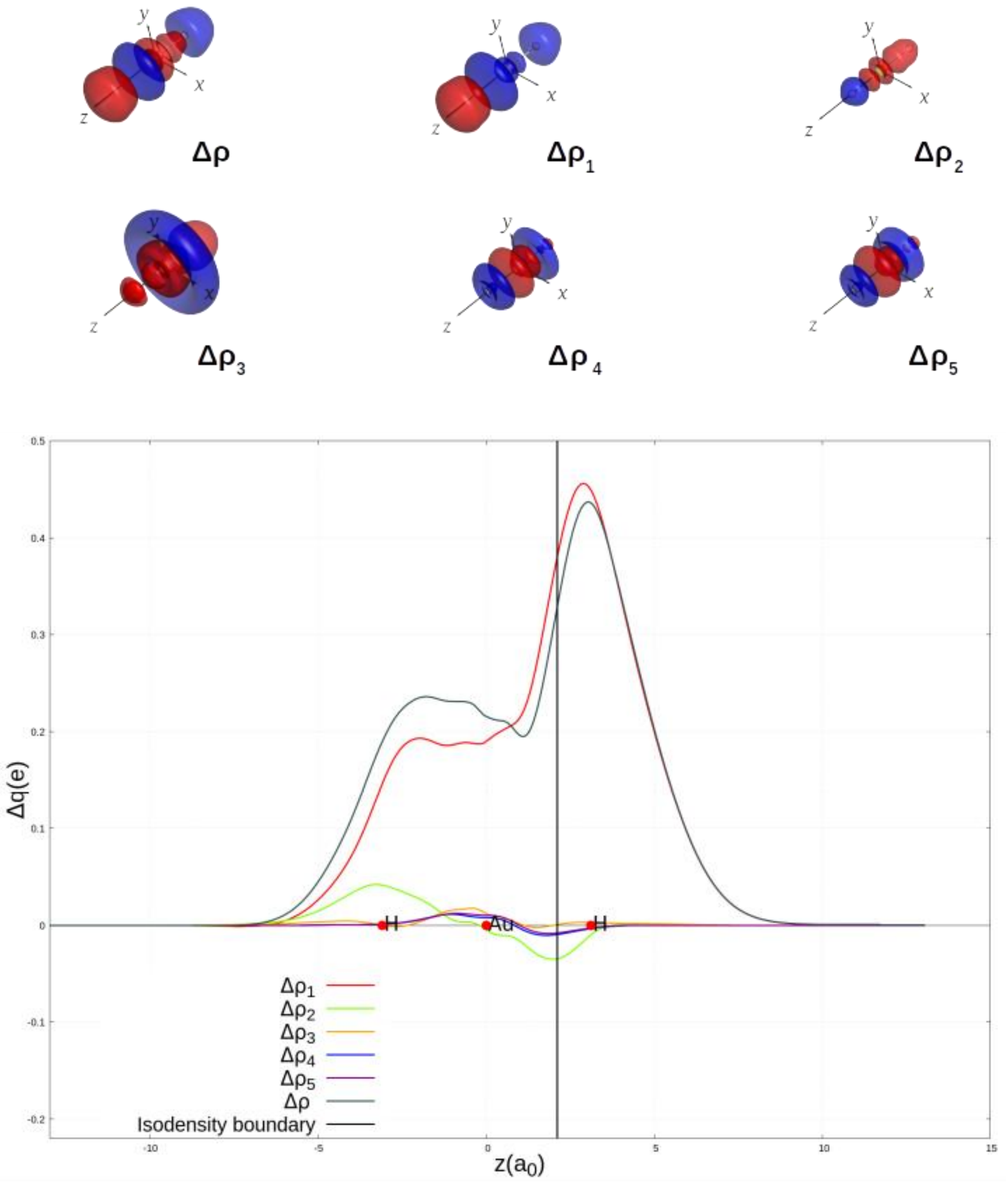

Figure S9. *Top:* SR-CD calculated isodensity surfaces of the total (Δρ) and of its first five NOCV components for the CuH_2^- complex (the isovalue is ±0.005 e a_0^{-3}). Blue regions indicate electron charge accumulation areas, whereas red regions indicate depletion areas.
Bottom: corresponding SR-CD curves for the CuH_2^- complex. Red dots indicate the position of the nuclei along the z axis. The vertical line marks the isodensity boundary.

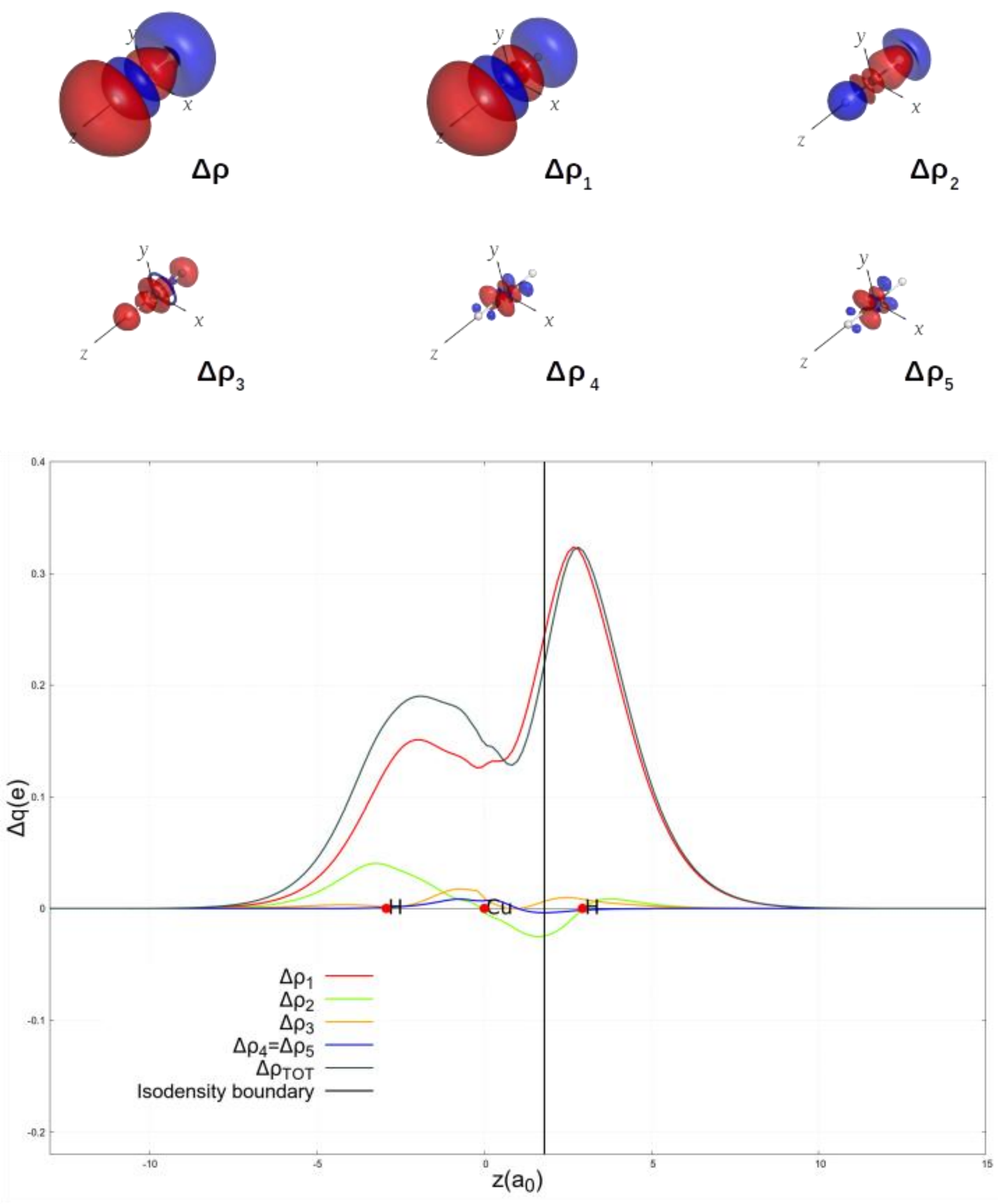

Figure S10. *Top:* SR-CD calculated isodensity surfaces of the total (Δρ) and of its first five NOCV components for the AgH_2^- complex (the isovalue is ±0.005 e a_0^{-3}). Blue regions indicateelectron charge accumulation areas, whereas red regions indicate depletion areas.
Bottom: corresponding SR-CD curves for the AgH_2^- complex. Red dots indicate the position of the nuclei along the z axis. The vertical line marks the isodensity boundary.

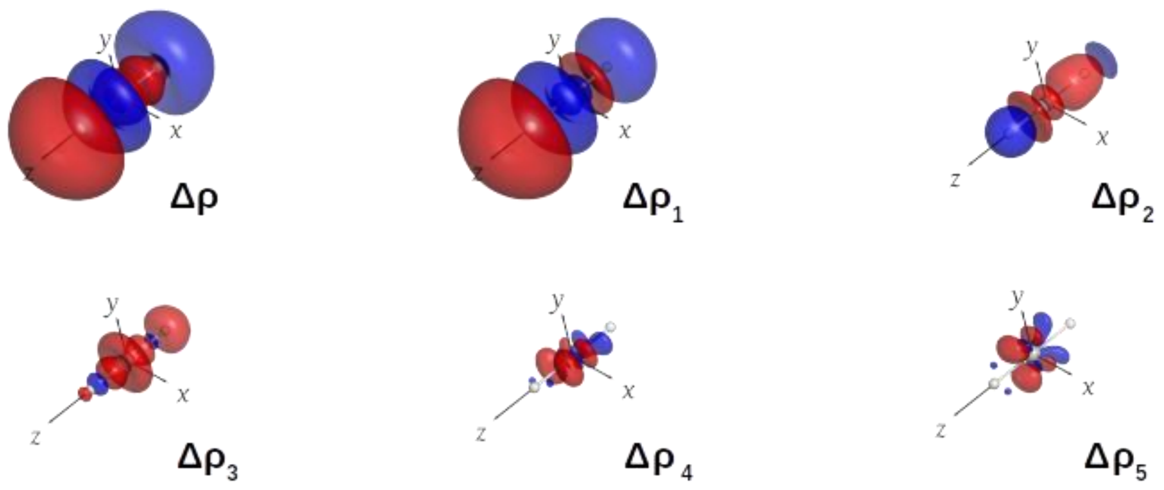

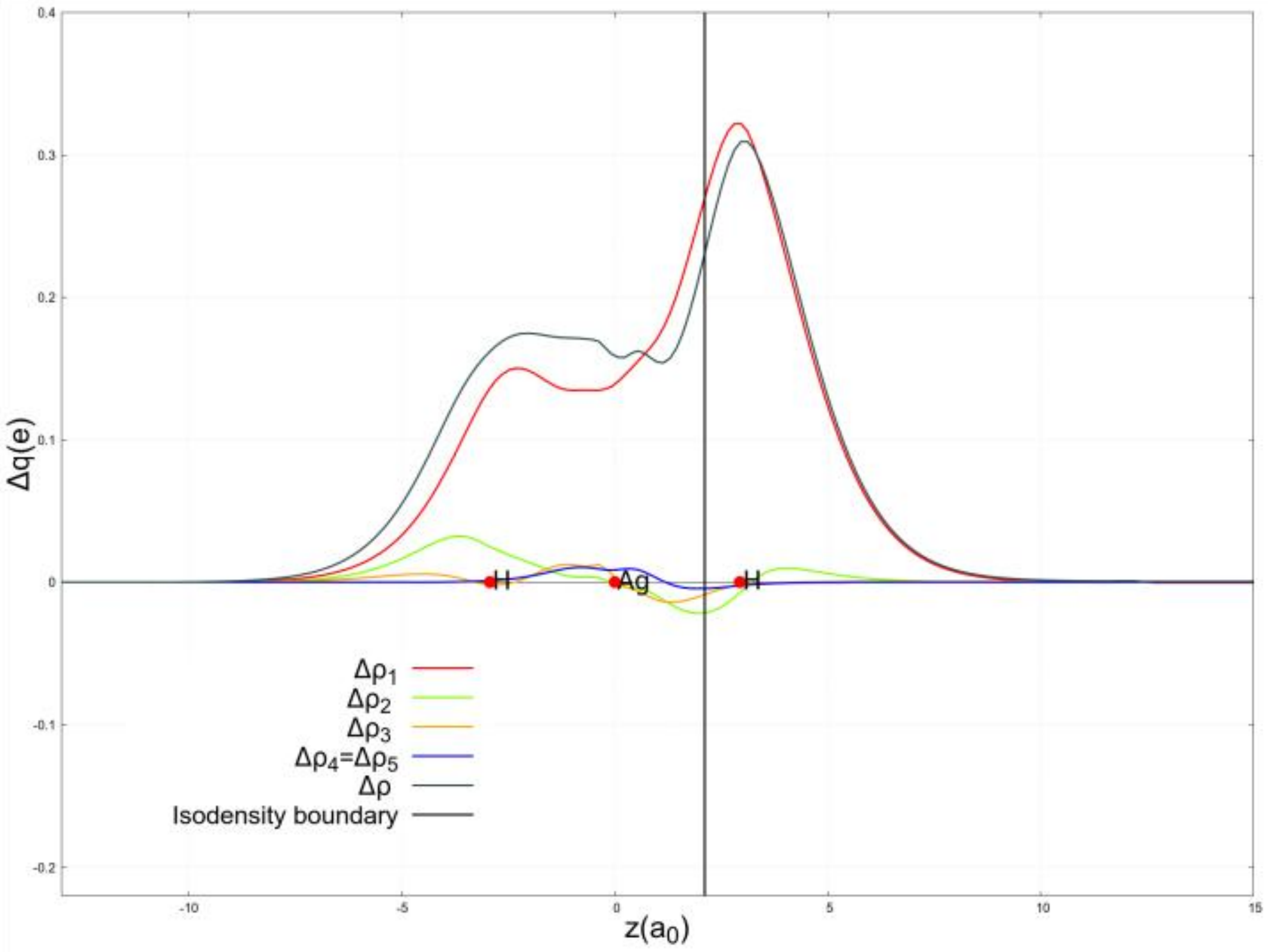

Figure S11. *Top:* SR-CD calculated isodensity surfaces of the total (Δρ) and of its first five NOCV components for the AuH_2^- complex (the isovalue is ±0.005 e a_0^{-3}). Blue regions indicate electron charge accumulation areas, whereas red regions indicate depletion areas.
Bottom: corresponding SR-CD curves for the AuH_2^- complex. Red dots indicate the position of the nuclei along the z axis. The vertical line marks the isodensity boundary.

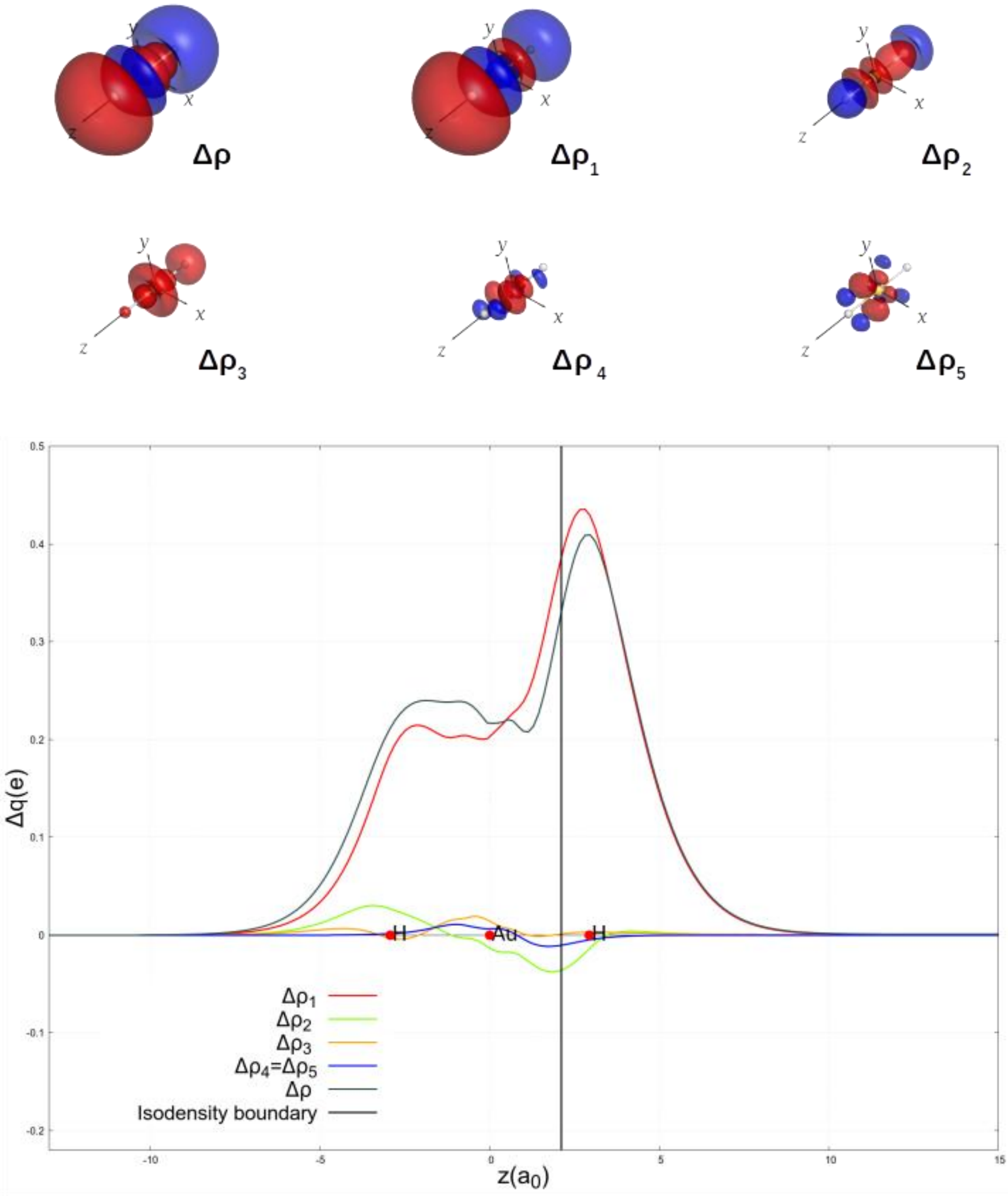

Figure S12. *Top:* SR-CD calculated isodensity surfaces of the total (Δρ) and of its first five NOCV components for the RgH_2^- complex (the isovalue is ±0.005 e a_0^{-3}). Blue regions indicate electron charge accumulation areas, whereas red regions indicate depletion areas.
Bottom: corresponding SR-CD curves for the RgH_2^- complex. Red dots indicate the position of the nuclei along the z axis. The vertical line marks the isodensity boundary.

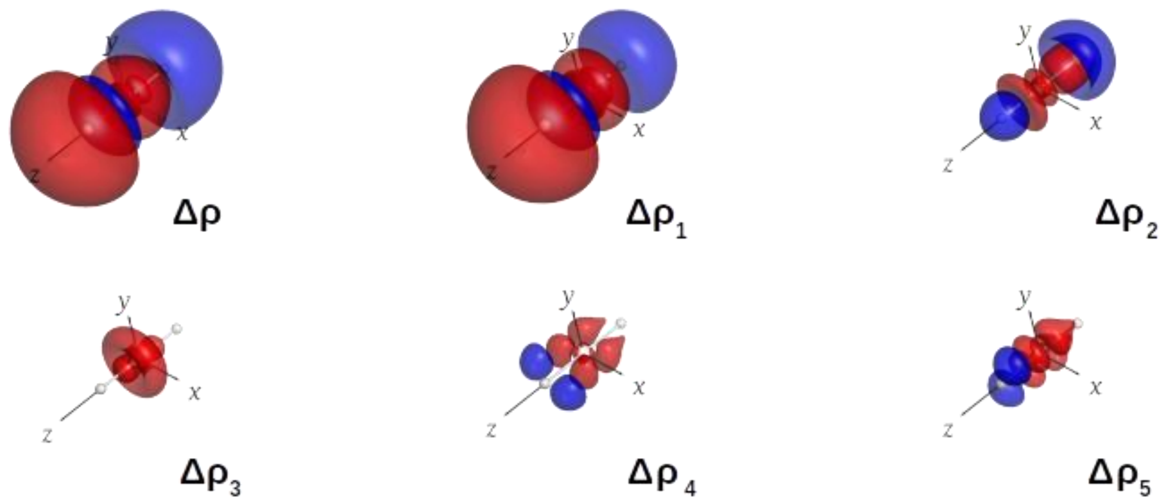

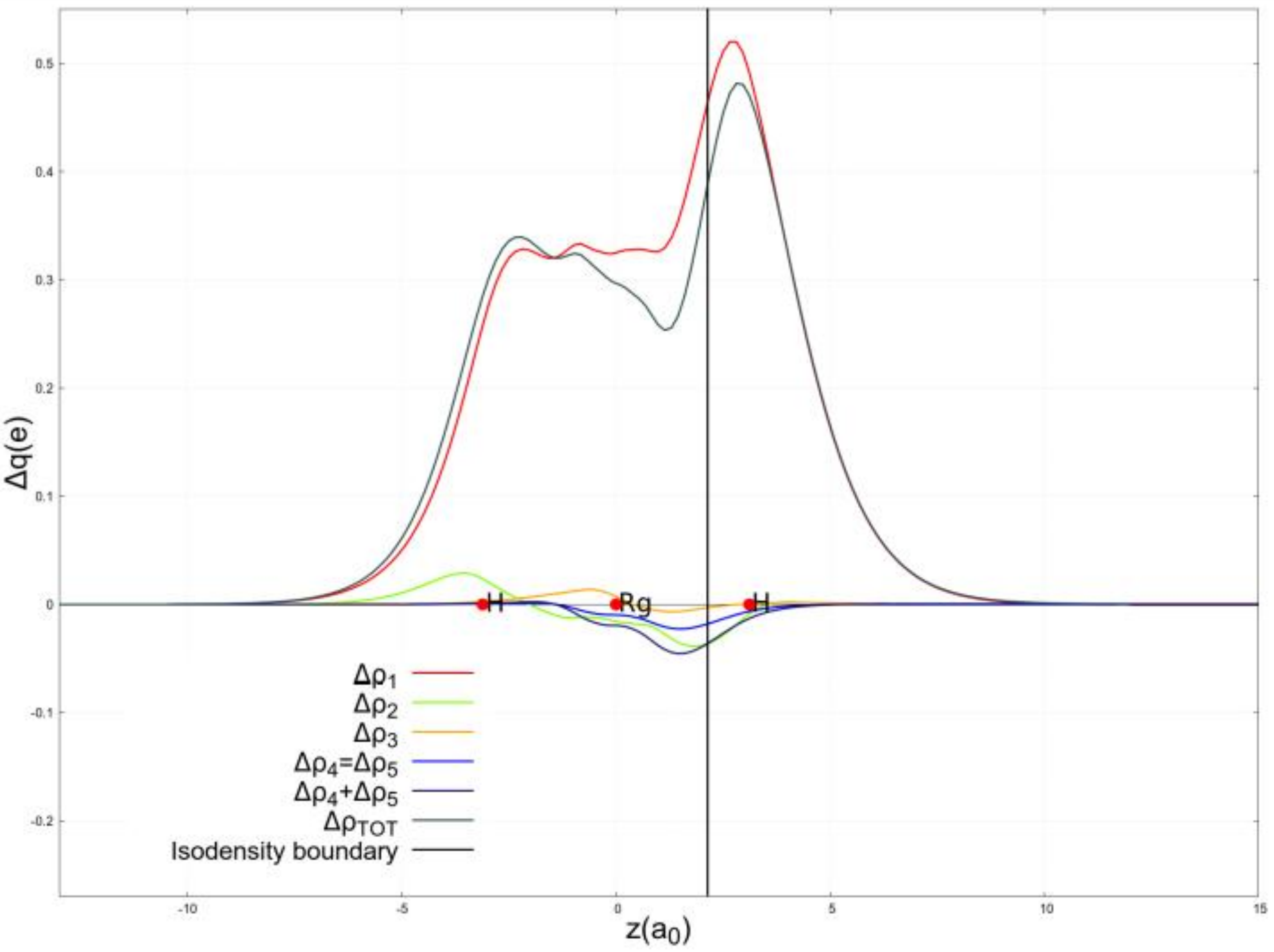

Table S5. SR-CD calculated charge Transfer (CT) values corresponding to NOCVs for MH_2^- complexes calculated by scalar relativistic Charge Displacement analysis (SR-CD).

**Complex**	**CT (e)**					
	**$\Delta\rho$**	**$\Delta\rho_1$**	**$\Delta\rho_2$**	**$\Delta\rho_3$**	**$\Delta\rho_4$**	**$\Delta\rho_5$**
**CuH_2^-**	0.219	0.244	-0.024	0.006	-0.004	-0.004
**AgH_2^-**	0.230	0.269	-0.021	-0.008	-0.004	-0.004
**AuH_2^-**	0.329	0.385	-0.036	-0.001	-0.010	-0.010
**RgH_2^-**	0.401	0.475	-0.034	-0.003	-0.019	-0.019

Figure S13. DKS-CD curves of the $\Delta\rho_1$ component (red line) and of parent NOCV-pairs densities $|\Phi_{-1}|^2$ (orange line) and $|\Phi_{+1}|^2$ (green line) for the CuH_2^- complex. The isodensity pictures (isovalue: ± 0.005 e a_0^{-3}) corresponding to the two parent densities have been also reported, where red and blue regions correspond to electron charge depletion and accumulation areas, respectively.

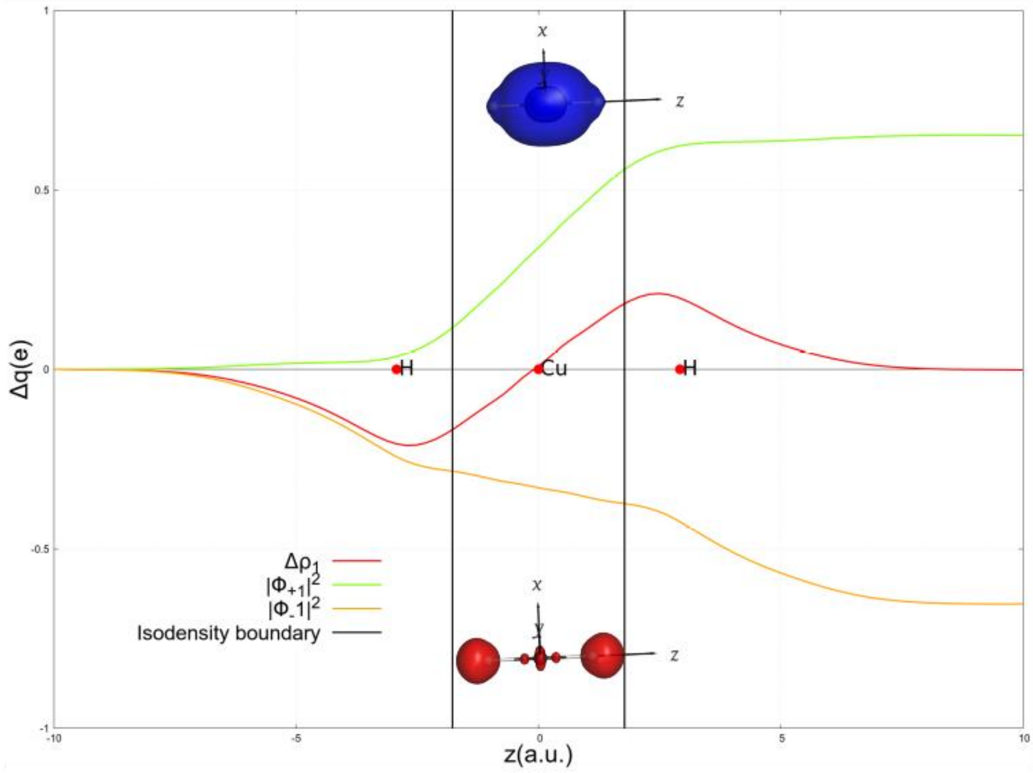

Figure S14. DKS-CD curves of the $\Delta\rho_1$ component (red line) and of parent NOCV-pairs densities $|\Phi_{-1}|^2$ (orange line) and $|\Phi_{+1}|^2$ (green line) for the AgH_2^- complex. The isodensity pictures (isovalue: ± 0.005 e a_0^{-3}) corresponding to the two parent densities have been also reported, where red and blue regions correspond to electron charge depletion and accumulation areas, respectively.

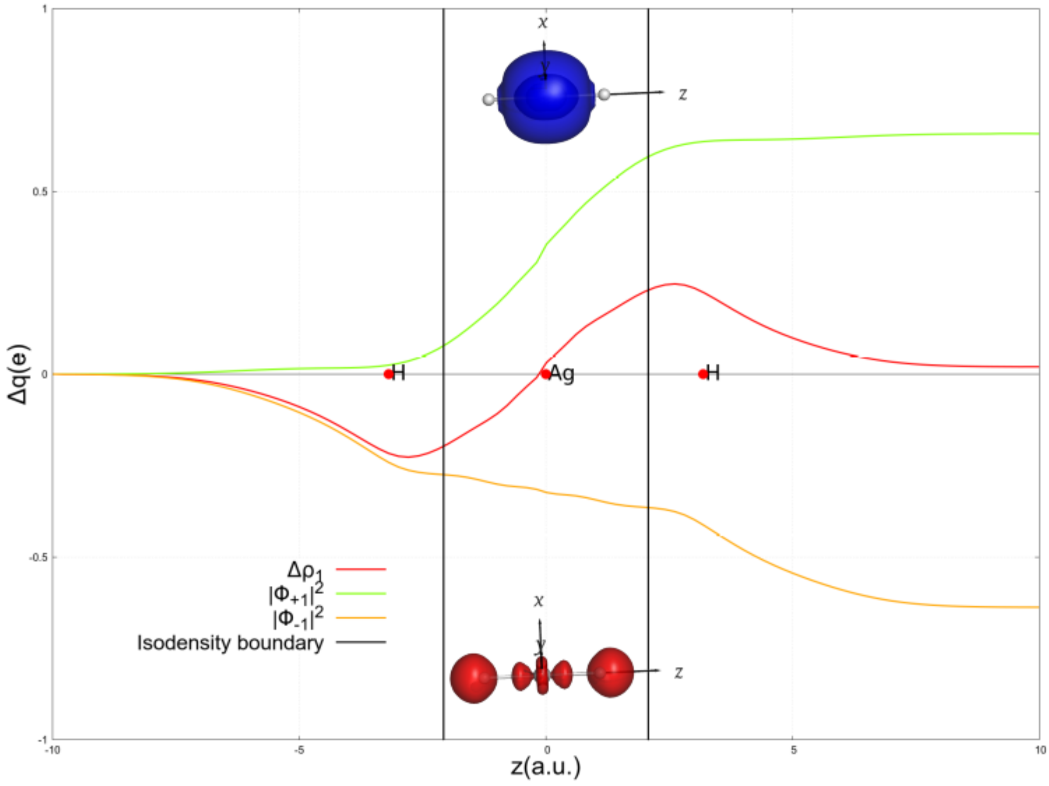

Figure S15. DKS-CD curves of the $\Delta\rho_1$ component (red line) and of parent NOCV-pairs densities $|\Phi_{-1}|^2$ (orange line) and $|\Phi_{+1}|^2$ (green line) for the AuH_2^- complex. The isodensity pictures (isovalue: ± 0.005 e a_0^{-3}) corresponding to the two parent densities have been also reported, where red and blue regions correspond to electron charge depletion and accumulation areas, respectively.

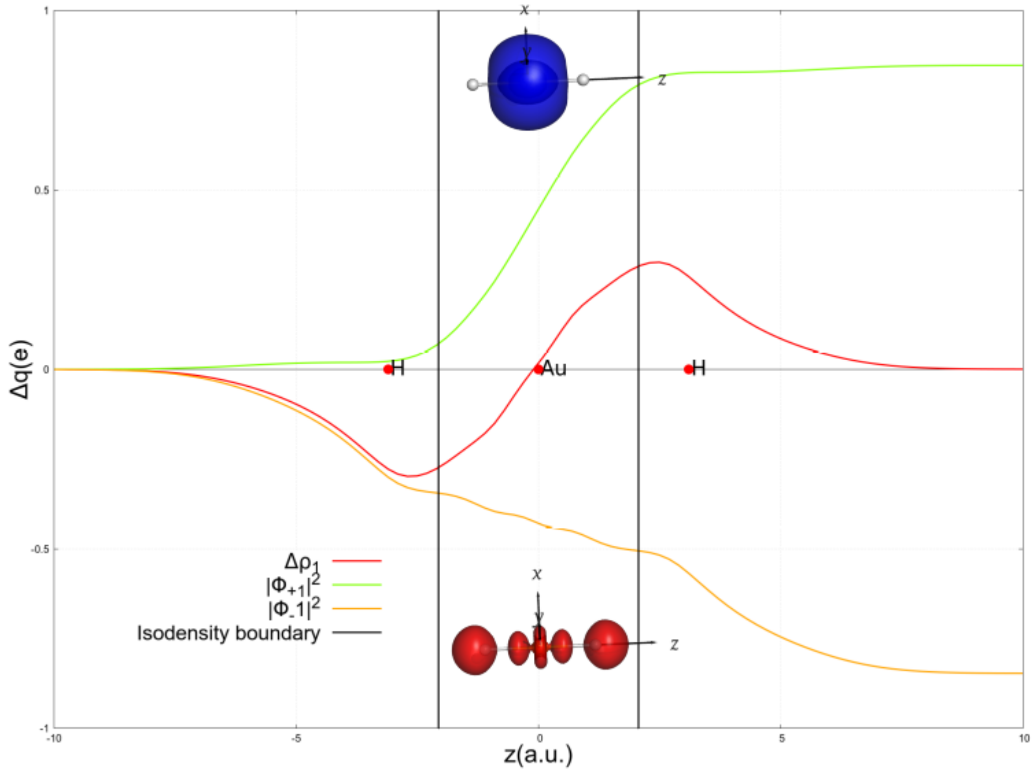

Figure S16. DKS-CD curves of the $\Delta\rho_1$ component (red line) and of parent NOCV-pairs densities $|\Phi_{-1}|^2$ (orange line) and $|\Phi_{+1}|^2$ (green line) for the RgH_2^- complex. The isodensity pictures (isovalue: ± 0.005 e a_0^{-3}) corresponding to the two parent densities have been also reported, where red and blue regions correspond to electron charge depletion and accumulation areas, respectively.

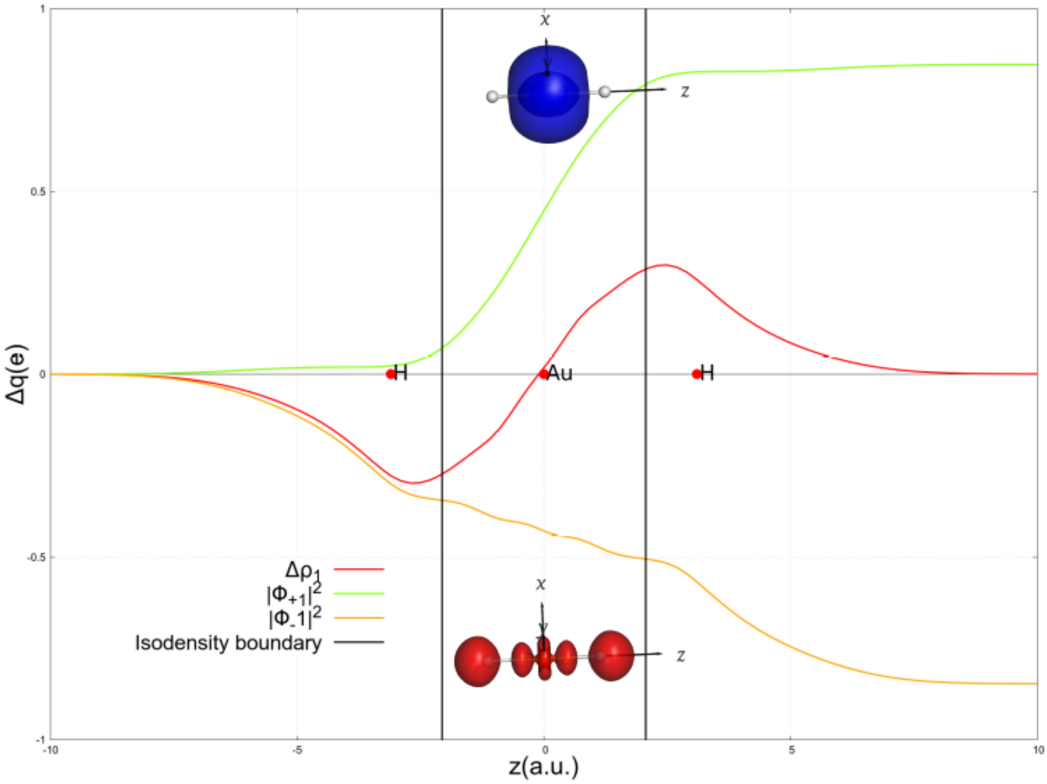

Table S6. Population of (n+1)s and nd orbitals of the metal atoms of the Group 11 anion dihydrides (n=4 for Cu, n=4 for Ag, n=5 for Au and n=6 for Rg) calculated by the means of non-relativistic (NR) and 4-component (4c) projection analysis.

**Atomic Orbital**	**Population**							
	**Cu (NR)**	**Cu (4c)**	**Ag (NR)**	**Ag (4c)**	**Au (NR)**	**Au (4c)**	**Rg (NR)**	**Rg (4c)**
**$nd_{5/2,1/2}$**		1.87		1.88		1.78		1.62
**$nd_{5/2,-3/2}$**		1.99		1.99		1.98		1.96
**$nd_{5/2,5/2}$**	9.79	2.00	9.82	2.00	9.74	2.00	9.68	1.99
**$nd_{3/2,1/2}$**		1.92		1.93		1.90		1.86
**$nd_{3/2,-3/2}$**		2.00		2.00		2.00		1.99
**$(n+1)s_{1/2}$**	1.11	1.15	1.03	1.11	1.15	1.40	1.15	1.68

Figure S17. Correlation between the populations of (n+1)s orbitals of the metal atom for Group 11 dihydrides (calculated at the 4-component level by the means of projection analysis) and the calculated extent of the sd hybridization (Δ_{HYBR}).

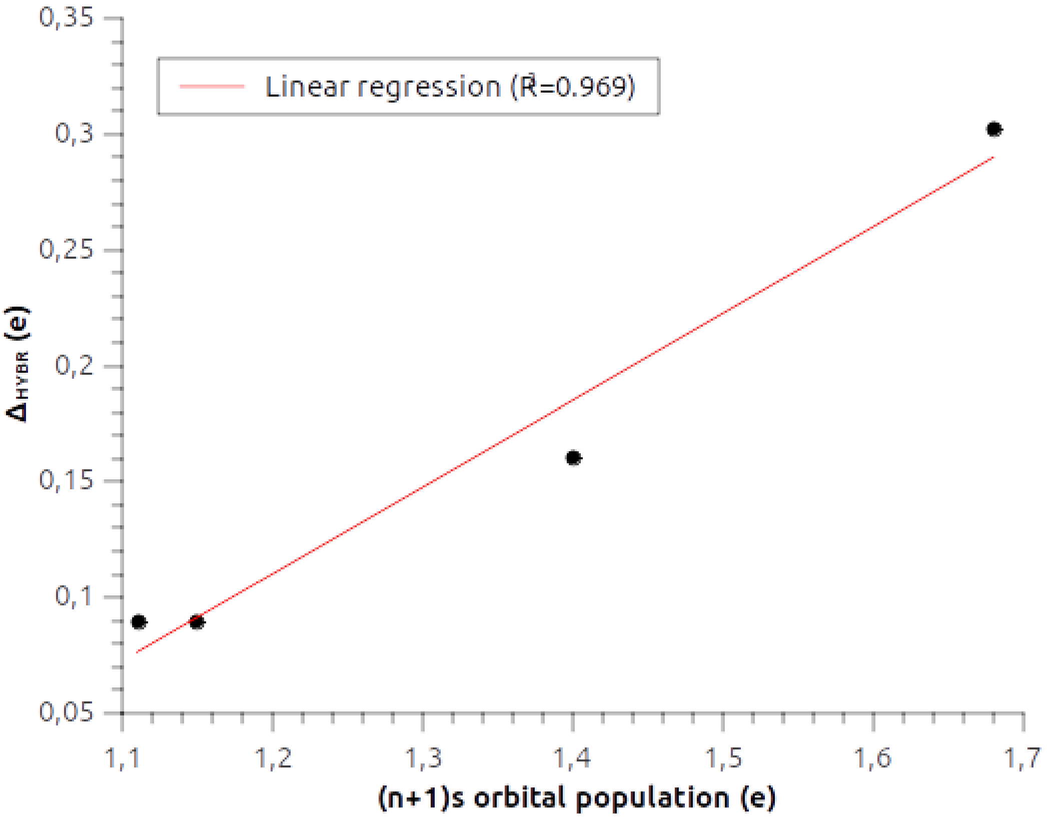

References

1. Andrews, L. & Wang, X. Infrared Spectra and Structures of the Stable CuH_2^-, AgH_2^-, AuH_2^-, and AuH_4^- Anions and the AuH_2 Molecule. *J. Am. Chem. Soc.* **125**, 11751–11760 (2003).
2. Liu, H.-T. *et al.* The electronic structure and chemical bonding in gold dihydride: $AuH_2^−$ and AuH_2. *Chem. Sci.* **3**, 3286–3295 (2012).
3. Xiong, X.-G. *et al.* On the gold–ligand covalency in linear $[AuX_2]^−$ complexes. *Dalton Trans.* **44**, 5535–5546 (2015).
4. Xu, C.-Q., Xiong, X.-G., Li, W.-L. & Li, J. Periodicity and Covalency of $[MX_2]^−$ (M = Cu, Ag, Au, Rg; X = H, Cl, CN) Complexes. *European Journal of Inorganic Chemistry* **2016**, 1395–1404 (2016).
5. Belpassi, L., Storchi, L., Quiney, H. M. & Tarantelli, F. Recent advances and perspectives in four-component Dirac–Kohn–Sham calculations. *Phys. Chem. Chem. Phys.* **13**, 12368–12394 (2011).
6. Quiney, H. M. & Belanzoni, P. Relativistic density functional theory using Gaussian basis sets. *J. Chem. Phys.* **117**, 5550–5563 (2002).
7. Quiney, H. M., Skaane, H. & Grant, I. P. Relativistic calculation of electromagnetic interactions in molecules. *J. Phys. B: At. Mol. Opt. Phys.* **30**, L829–L834 (1997).
8. Belpassi, L., Storchi, L., Tarantelli, F., Sgamellotti, A. & Quiney, H. M. Parallelization of a relativistic DFT code. *Future Generation Computer Systems* **20**, 739–747 (2004).
9. Belpassi, L., Tarantelli, F., Sgamellotti, A. & Quiney, H. M. Computational strategies for a four-component Dirac–Kohn–Sham program: Implementation and first applications. *J. Chem. Phys.* **122**, 184109 (2005).
10. Dyall, K. G. Relativistic double-zeta, triple-zeta, and quadruple-zeta basis sets for the 5d elements Hf–Hg. *Theor Chem Acc* **112**, 403–409 (2004).
11. Dyall, K. G. Relativistic double-zeta, triple-zeta, and quadruple-zeta basis sets for the 4d elements Y–Cd. *Theor Chem Acc* **117**, 483–489 (2007).
12. Dyall, K. G. Relativistic double-zeta, triple-zeta, and quadruple-zeta basis sets for the 6d elements Rf–Cn. *Theor Chem Acc* **129**, 603–613 (2011).
13. Dyall, K. G. Relativistic double-zeta, triple-zeta, and quadruple-zeta basis sets for the light elements H–Ar. *Theor Chem Acc* **135**, 128 (2016).
14. Grant, I. P. & Quiney, H. M. Rayleigh-Ritz approximation of the Dirac operator in atomic and molecular physics. *Phys. Rev. A* **62**, 022508 (2000).
15. Belpassi, L., Tarantelli, F., Sgamellotti, A. & Quiney, H. M. Electron density fitting for the Coulomb problem in relativistic density-functional theory. *J. Chem. Phys.* **124**, 124104 (2006).
16. Koster, A. M. & Salahub, D. R. deMon2k, version 4; Cinvestav, Mexico City, 2016. https://hadoop.apache.org.
17. De Santis, M., Belapassi, L., Tarantelli, F. & Storchi, L. Relativistic quantum chemistry involving heavy atoms. *Rend. Fis. Acc. Lincei* **29**, 209–217 (2018).

18. Perdew, J. P., Burke, K. & Ernzerhof, M. Generalized Gradient Approximation Made Simple. *Phys. Rev. Lett.* **77**, 3865–3868 (1996).
19. Huber, K. P. & Herzberg, G. *Molecular Spectra and Molecular Structure*. (Van Nostrand, Princeton, 1979).
20 MacAdam, K. B.; Dyubko, S. F.; Efremov, V. A.; Gerasimov, V. G.; Kutsenko, A. S. Laser-Microwave Spectroscopy of Cu I Atoms in S, P, D, F and G Rydberg States. *J. Phys. B: At. Mol. Opt. Phys.* **2009**, *42* (16), 165009.
